# Annotation and Analysis of 3902 Odorant Receptor Protein Sequences from 21 Insect Species Provide Insights into the Evolution of Odorant Receptor Gene Families in Solitary and Social Insects

**DOI:** 10.3390/genes13050919

**Published:** 2022-05-20

**Authors:** Pablo Mier, Jean-Fred Fontaine, Marah Stoldt, Romain Libbrecht, Carlotta Martelli, Susanne Foitzik, Miguel A. Andrade-Navarro

**Affiliations:** 1 Institute of Organismic and Molecular Evolution (iomE), Faculty of Biology, Johannes Gutenberg University Mainz, Hanns-Dieter-Hüsch-Weg 15, 55128 Mainz, Germany; fontaine@uni-mainz.de (J.-F.F.); mastoldt@uni-mainz.de (M.S.); romain.libbrecht@gmail.com (R.L.); foitzik@uni-mainz.de (S.F.); andrade@uni-mainz.de (M.A.A.-N.); 2 Institute of Developmental Biology and Neurobiology (iDN), Faculty of Biology, Johannes Gutenberg University Mainz, Hanns-Dieter-Hüsch-Weg 15, 55128 Mainz, Germany; cmartell@uni-mainz.de

**Keywords:** odorant receptor, machine learning, chemical binding, insects

## Abstract

The gene family of insect olfactory receptors (ORs) has expanded greatly over the course of evolution. ORs enable insects to detect volatile chemicals and therefore play an important role in social interactions, enemy and prey recognition, and foraging. The sequences of several thousand ORs are known, but their specific function or their ligands have only been identified for very few of them. To advance the functional characterization of ORs, we have assembled, curated, and aligned the sequences of 3902 ORs from 21 insect species, which we provide as an annotated online resource. Using functionally characterized proteins from the fly *Drosophila melanogaster*, the mosquito *Anopheles gambiae* and the ant *Harpegnathos saltator*, we identified amino acid positions that best predict response to ligands. We examined the conservation of these predicted relevant residues in all OR subfamilies; the results showed that the subfamilies that expanded strongly in social insects had a high degree of conservation in their binding sites. This suggests that the ORs of social insect families are typically finely tuned and exhibit sensitivity to very similar odorants. Our novel approach provides a powerful tool to exploit functional information from a limited number of genes to study the functional evolution of large gene families.

## 1. Introduction

Odorant receptors (ORs) constitute the largest family of chemoreceptors expressed in the membranes of olfactory sensory neurons in insects. The insect odorant receptor gene family is an evolutionary novelty in the ancestor of all insects [1], likely an adaptation to sensory perception in terrestrial life. Insects use ORs to perceive sexual pheromones, food sources, including nectar-providing flowers, and, importantly, for social communication [2,3,4,5].

A rapid expansion of chemoreceptors, especially ORs, accompanied the repeated transition from a solitary to a social lifestyle in Hymenoptera [6,7]. The ecological success of social insects is based on their ability to form complex cooperative societies, which in turn was only made possible by their sophisticated chemical communication [8,9,10]. Particularly fascinating and diverse communication mechanisms are found in ants. Ants use secretions from 70 different glands to exchange information with their nestmates and also signal their colony affiliation, fertility, and caste membership via a complex mixture of long-chain hydrocarbons on their cuticle (CHCs) [11]. As in all other insects, the antenna is the primary organ for olfactory perception, and ants can express up to 500 different ORs in olfactory sensory neurons [12]. The connection between sociality and odorant receptor repertoire in ants is furthermore supported by experimental studies showing that the impairment of specific receptors affects social behavior [13,14], and by the finding that the partial loss of social behaviors in social parasites was accompanied by a loss of OR genes [15,16]. The 9-exon subfamily shows a particularly strong signal of expansion and association with the switch to sociality and social communication and this not only in the ants [17,18,19,20,21], but also in the social wasps [5]. As some 9-exon ORs bind multiple ligands and some bind the same [22,23], it has been suggested that this subfamily distinguishes odors using combinatorial coding [5,24]. Together with the aforementioned expansion of this subfamily, this may allow some insects, including ants, to discriminate between a wide variety of odors. Therefore, this OR subfamily in particular is a prime candidate for understanding how advances in chemical communication have led to the formation of eusocial societies in ants. While the specificity and tuning of the different ORs have been well studied in dipteran model species, such as *Drosophila melanogaster* and *Anopheles gambiae* [25,26,27], it remains largely unclear to which chemicals the extremely high number of ORs in social insects respond to [23]. This knowledge would be necessary to make predictions about the trajectory leading to the evolution of eusociality in insects.

Our aim is to extend the functional characterization of OR protein families to facilitate the generation of functional and evolutionary hypotheses. To achieve this, we use a machine learning approach. Machine learning has already been used in the field of insect ORs to identify ligands for specific ORs (e.g., [28,29]). Here, we use machine learning to evaluate the power of particular amino acid positions in 3902 OR sequences from 21 species to predict responses to chemicals according to available experimental data from three well-studied insects, the dipterans *Anopheles gambiae* [26] and *Drosophila melanogaster* [27], and the ant *H. saltator* [23]. Amino acids with predictive power were then mapped to 3D positions using as a template the only solved structure of a protein from the OR family, the Orco protein from the parasitic fig wasp *Apocrypta bakeri* [30]. Independent of the machine learning approach, we used sequence similarity to group the OR families of 21 insect species into clusters expected to have similar biological functions across species. We annotated these clusters according to their evolutionary expansion, taxonomic specificity, and conservation of their predicted binding sites to find modes of evolution associated with the emergence of biological and molecular function.

Our approach provides a way to transfer information between thousands of ORs already considered and allows for the extension of this information either to individual ORs from genomes not yet included in our resource, or potentially by including relevant OR datasets from complete genomes as well as new functional profiles, as needed. This approach can potentially be applied to other large families of paralogues. Analysis of these large families should allow us to understand how gene duplications drive the emergence of new functions.

## 2. Materials and Methods

### 2.1. Sequence Data Retrieval, Curation and Alignment

We obtained the annotated odorant receptor (OR) protein sequences from the following 21 insect species, including 8 ant species, 2 social bee species and 11 solitary insects from damsel flies to flies (Table 1): *Drosophila melanogaster* [31], *Anopheles gambiae* [26], *Apis mellifera*, *Solenopsis invicta*, *Nasonia vitripennis* and *Ooceraea biroi* [12], *Pogonomyrmex barbatus* [32], *Atta cephalotes* and *Acromyrmex echinatior* [18], *Camponotus floridanus* and *Harpegnathos saltator* [17], *Linepithema humile* [6], *Blattella germanica* [33], *Calopteryx splendens* [34], *Bombus terrestris* [35], *Tribolium castaneum* [36], *Cloeon dipterum* [37], *Manduca sexta* [38], *Pediculus humanus* [39], *Acyrthosiphon pisum* and *Aphis glycines* [40].

For manual curation, we first aligned all retrieved raw sequences of each species independently. Sequences identified as pseudogenes or fragments were removed, as well as sequences that had large and unique insertions and deletions. Next, the entire dataset of 3902 curated OR proteins was aligned using MAFFT v7.453 with default parameters [41] (Supplementary File S1). The complete taxonomic lineage from each of the species was obtained from the NCBI resource Common Taxonomy Tree [42]. We resolved the phylogenetic relationships in ants with information from Borowiec et al., 2020 [43].

### 2.2. Clustering of OR Proteins

Protein sets were clustered using a method designed to infer orthologous groups across species (OrthoFinder v2.3.12 with default parameters [44]). To be able to associate the clusters with previously identified odorant receptor subfamilies, we used the mapping of ORs to subfamilies in *C. floridanus* and *H. saltator* provided by [17], and the ones from *A. echinatior* and *A. cephalotes* provided by [18]. First, for each of the four species, we extracted the information which OR belonged to which cluster. Then we assigned the ORs to their respective subfamily. For *A. cephalotes*, we renamed the subfamily “unassigned N???” to “unassigned” to match the unassigned records for the other species. Similarly, missing information on the subfamily was designated as “unassigned”. In addition, an OR with subfamily “0” was noted for *A. echinatior* (typo in the original publication), and we changed this to “O”.

### 2.3. Machine Learning Approach

We transformed the multiple sequence alignment of all OR sequences into a table to be used in the machine learning procedure, showing the amino acids (cells of the table) of the proteins (rows) at each position of the alignment (columns or machine learning variables). A machine learning variable is defined here as a position in the alignment and a machine learning feature as an amino acid at a particular position. Additional columns contain numerical experimental chemical response data for some proteins from datasets of three species: *D. melanogaster* [27], *A. gambiae* [26], and *H. saltator* [23]. The three of them together include chemical-response data for a total of 672 chemicals. For the machine learning training phase, 8 out of 672 chemicals with the highest number of chemical effect data (>100 values in the union of the 3 data sets) and 494 out of 3902 proteins (associated with at least one chemical effect value) were selected. The eight selected chemicals have the following registry numbers and IUPAC names (common names in parentheses): 108-94-1 cyclohexanone, 431-03-8 butane-2,3-dione (diacetyl), 67-64-1 propan-2-one (acetone), 110-43-0 heptan-2-one (2-heptanone), 6728-26-3 (E)-hex-2-enal (trans-2-hexenal), 119-36-8 methyl 2-hydroxybenzoate (methyl salicylate), 105-87-3 [(2E)-3,7-dimethylocta-2,6-dienyl] acetate (geranyl acetate), 3391-86-4 oct-1-en-3-ol (vinyl amyl carbinol).

For each of the eight chemicals within each dataset, chemical-response values were binarized by setting a value greater than the 75th percentile to one to represent positive response, 0 otherwise to represent lack of response (Supplementary File S2; not-tested combinations simply lack values). For each chemical-dataset pair (3 datasets and 8 chemicals: 24 pairs), a random forest (RF) model based on 500 trees was trained to predict chemical-response values using the machine learning variables. Only proteins associated with a chemical-response value were used in the training set: 47, 45 and 23 ORs from *D. melanogaster*, *A. gambiae* and *H. saltator*, respectively. Furthermore, near zero-variance variables were filtered out. The analysis was implemented in R with the caret and randomForest packages (the optimal mtry parameter, defining the optimal number of predictors for split, was defined by grid search during training phase; tested mtry values: 20, 50 and 100). A model’s performance was derived from internal cross-validations (10-fold cross-validations repeated 10 times) and model measures of feature importance were scaled by the caret package to have a maximum value of 100. Performance during the cross-validations is reported as area under ROC curves, F1 score, sensitivity, or precision.

### 2.4. Computation of Sequence Conservation

To measure the sequence conservation of the ORs in a cluster, each position in the alignment was given a conservation score, which is simply the occurrence of the most frequent residue at the position for the ORs in the cluster: a conservation score of one indicates a fully conserved position, while highly variable positions receive scores close to zero. We then calculated for each cluster a background sequence conservation, i.e., the average conservation value of all residues in the sequence, and for comparison a predictive residue conservation, i.e., the average conservation of the predictive residues selected by machine learning. In general, we restricted this calculation to clusters with five or more ORs.

## 3. Results

### 3.1. Collection and Curation of Insect Odorant Receptor Proteins

**Figure 1:**
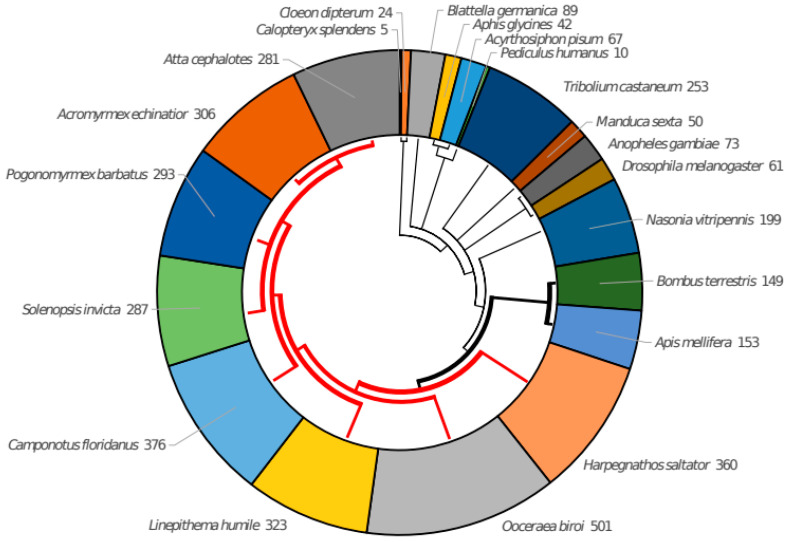
Number of curated ORs for each species. The tree represents the phylogenetic relationships between the species. Thick branches indicate social insects and red color indicates ants. While all social insects have more than 100 ORs, it is the case of only 2 non-social insects out of 11 (the wasp *N. vitripennis* and the beetle *T. castaneum*).

We first collected OR protein sequences from a variety of insect species. We manually examined published data for 21 insect species with fully sequenced genomes (see Methods). Given the dynamic nature of sequencing new genomes, it seems necessary to update such a collection, as it is not only of interest to other researchers in the field of OR evolution, but also to computational biologists developing methods for function prediction using machine learning or other approaches. For these reasons, we have developed a special repository called iOrME (insect Odorant Receptors Molecular Evolution), which collects all raw and curated OR datasets as well as taxonomic information on the insect species we use. It is available at http://cbdm-01.zdv.uni-mainz.de/~munoz/iorme/ (accessed on 21 April 2022), with no restrictions for users. For this first version of iOrME (v1.0) we collected a raw dataset of 4708 OR sequences. The dataset also contained fragments and pseudogenes. After manual curation, we ended up with a core dataset of 3902 OR proteins (see Methods for details; Figure 1; Table 1). Some sets needed more attention than others. For example, while for the leafcutter ant *Atta cephalotes* we removed 35% of the original sequences (from 434 to 281 proteins) and 52% for the mayfly *Cloeon dipterum* (from 50 to 24 proteins), the 61 well-established OR proteins from *D. melanogaster* remained, as well as the 293 proteins from the red harvester ant *Pogonomyrmex barbatus*.

### 3.2. The Taxonomic Distribution of ORs in Clusters Shows Taxa-Specific Expansions

We performed sequence clustering of the 3902 OR sequences using a method that inferred orthologous groups in different species (see Methods). Our aim is to assess the relationship between the evolutionary history of these OR subfamilies and their ligand-binding properties. Clustering revealed a total of 206 clusters, 40 of which consisted of a single protein (singletons) and would be expected to correspond to very species-specific functions (Supplementary Files S3 and S4; FASTA files containing the sequences of each cluster are available for download in iOrME). The largest clusters largely correspond to the subfamilies of ant ORs previously described in [17] based on genome organization, and then expanded in [18] (Supplementary Figure S1).

**Figure 2:**
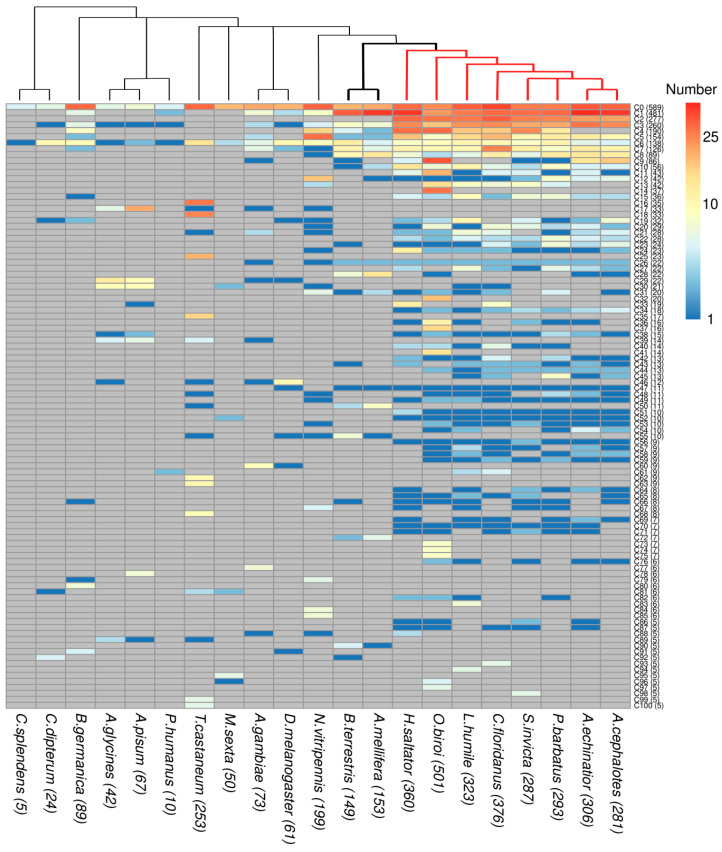
Species distribution of OR proteins per cluster. For each cluster, the number of ORs from each species is indicated. Gray cells indicate no OR from a species in a cluster. Only clusters with five or more proteins are shown. The tree above shows the phylogenetic relations of the 21 insect species: bold and red branches indicate social insects and ants, respectively. The total number of ORs per cluster and per species are shown in parenthesis.

Next, we examined the species distribution of the ORs (Figure 2). The taxonomic distribution of ORs varies widely across clusters, reflecting the complicated evolutionary history of this family. Only two groups, C0 and C6 (with 589 and 138 sequences, respectively), contain at least one protein from all 21 species. C6 includes Orco, one of the ancestral proteins of the family, which is highly conserved across species and forms a heteromeric cation channel with an OR subunit [45,46]. Interestingly, one of the most populated clusters, C2 with 277 sequences, has ORs from all ants and only from the ants. It is comprised mostly of 9-exon ORs (Supplementary Figure S1), a subfamily known to be expanded in ants and paper wasps (not included in our dataset) [5,17,18,19,21]. Cluster C22 is ant specific too, in this case composed only of ORs from the V subfamily, also shown to be expanded in ants [18]. Among the single species clusters, C14 (37 sequences) and C16 (35 sequences), from the ant *O. biroi* and the beetle *T. castaneum*, respectively, stand out as the most populated.

**Figure 3:**
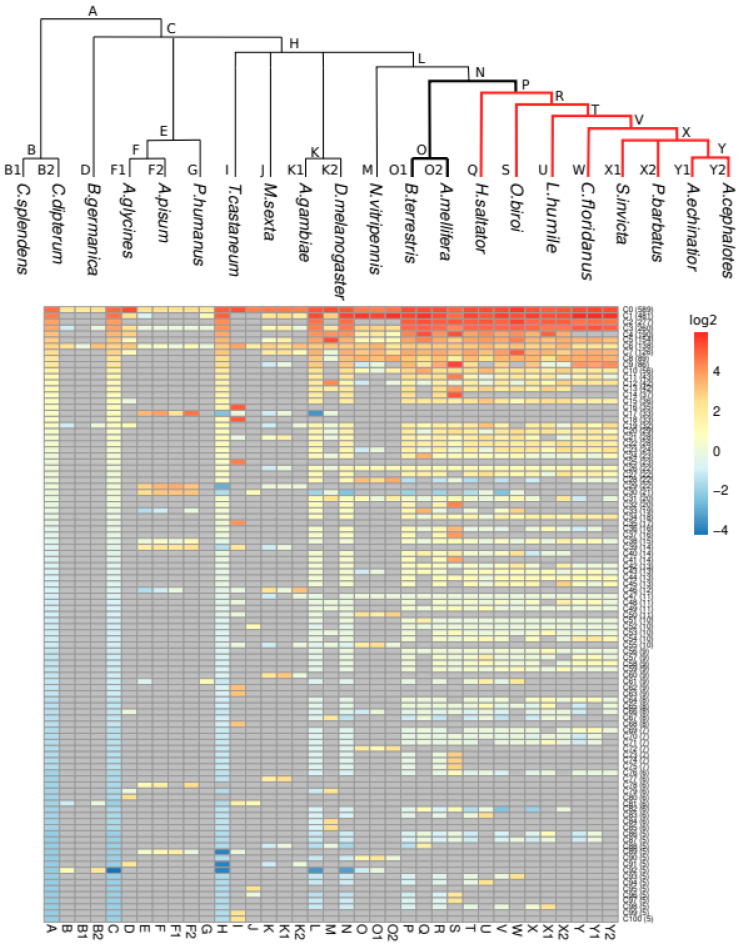
Enrichment of taxon-related ORs per cluster. For each cluster and each taxon, the log2-transformed ratio between the number of taxon-related OR proteins and the number of species in the taxon is shown. A positive value denotes a higher number of taxon-related proteins from the cluster than the number of species in the taxon. A negative value denotes a lower number of taxon-related proteins from the cluster than the number of species in the taxon. Each taxon is defined by a letter, depicted in the phylogenetic tree above and described in Table 2. In bold, social insects. In red, ants. Only clusters with five or more proteins are shown.

Analysis of these profiles can be used to investigate gene loss when a cluster contains members from all but one or a few species in a taxonomic group. One such example is C40, which contains 14 OR genes from six of the eight ant species considered in our study, but is absent from *A. echinatior* and *A. cephalotes*, suggesting that this OR cluster has been lost from the fungus-growing ants (Attini). Some clusters contain ORs from very different species, but they do not expand. An extreme example is C47, which contains 11 sequences from 11 species (*D. melanogaster* and the 10 Aculeata considered in this study, which include the ant and bee species). To evaluate the existence of taxa-specific expansions within our clusters, we measured the enrichment of taxon-specific ORs in each cluster, by computing for each cluster and taxon the log2-transformed ratio between the number of sequences from the given taxon and the number of species in it (Figure 3; for definitions, see Table 2). Using this representation, we can find a number of clusters that reflect taxa-specific expansions in Hemiptera coupled to gene loss in ants: C17, C29 and C30. Many of these sequences were noted in [47] as the “Clade A” of *A. pisum*-specific recent and rapid OR expansion. We note also C28 (22 sequences) as the cluster with the most relevant Apoidea-specific expansion (13 sequences from *A. mellifera* and 6 from *B. terrestris*).

### 3.3. Prediction of OR Amino Acid Residues Important for Chemical Binding

Insect odorant receptors bind chemicals to trigger neuronal activity essential for odorant perception [12]. While the functional information on OR family chemical binding is very limited, the multiple sequence alignment of the family contains a wealth of information on the variability of residues at positions that interact with odorants. We hypothesized that the availability of datasets containing a profile of neuronal responses of a large number of ORs to standard chemicals would allow a machine learning approach to identify positions in the alignment corresponding to residues involved in molecular recognition of odorants. Such an approach is supported by work suggesting that the OR family has a general common mechanism of interaction with odorants according to structural analysis [48].

**Figure 4:**
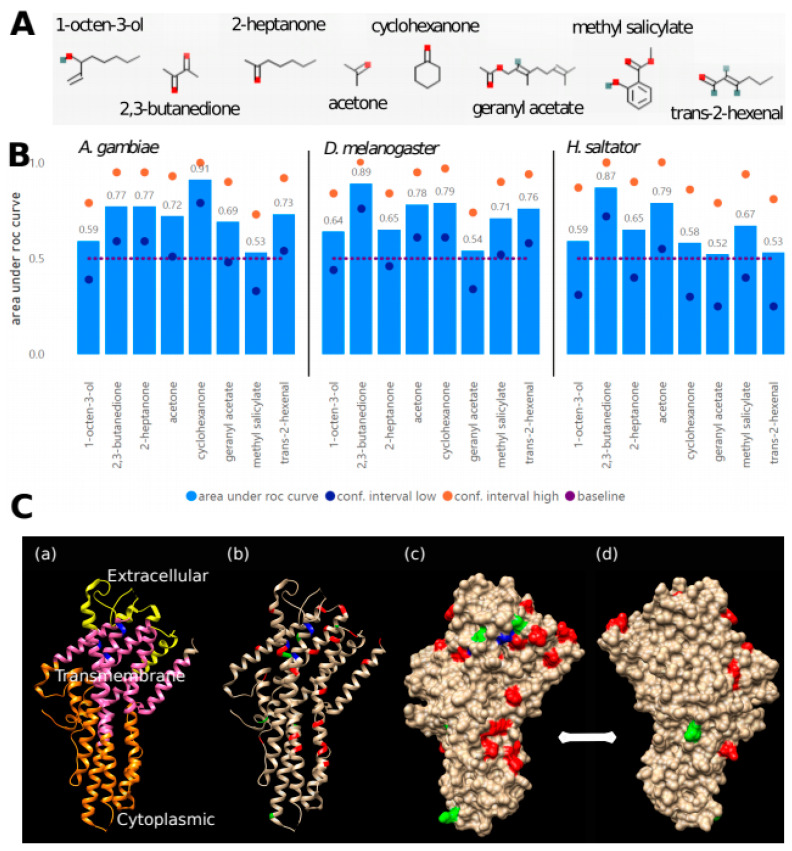
Detection of amino acid positions predictive for chemical binding. (**A**) Chemicals used in the training of the machine learning approach (see Methods for registry names and IUPAC names). (**B**) Cross-validation performance of the machine learning models was evaluated by area under ROC curves (true positive rate against false positive rate at variable thresholds; best curve evaluating either the positive or the negative class) during 10-fold cross-validations repeated 10 times. A random forest model of 500 trees was trained for each species–chemical pair to predict the binding of the chemical to the species-related OR proteins. Low and high boundaries of the 95% confidence interval and baseline (0.5 = random classification) are shown. (**C**) Mapping predictive features on 3D structure: (**a**) 3D structure of the Orco protein from the parasitic fig wasp *Apocrypta bakeri* (PDB:6C70) [33]; (**b**–**d**) Top 10 positions predicted for any of the three datasets are indicated in red. Positions detected among the top 100 in the three datasets (6 positions) are indicated in green. Positions indicated in blue (*A. bakeri* amino acid positions 143, 149–150, 202) were mapped from positions whose mutations were experimentally shown to modify ligand detection [49,50,51]; (**d**) shows the molecule rotated 180° along the vertical axis.

We used previously published data of the OR response to panels of chemicals from three insect species: *D. melanogaster* (48 ORs, 618 chemicals) [27], *A. gambiae* (50 ORs, 110 chemicals) [26] and *H. saltator* (25 ORs, 37 chemicals) [23]. To identify and characterize amino acid positions and residues potentially important for the binding, we used both the available chemical binding information of eight selected chemicals (Figure 4A) and the OR sequence alignment to train machine learning models of prediction (see Methods for details). Classification performance varied across models during cross-validations, with often higher sensitivity than precision (Figure 4B; Supplementary Figure S2; Supplementary File S5). Predictions for some chemicals (e.g., 2,3-butanedione) are clearly better than for others. We also observe differences between the datasets with generally worse predictions for the ant dataset, which could be due to the selection of chemicals, some of which might be unimportant for ants.

After analyzing 2892 distinct positions in the alignment, we obtained the importance of each amino acid at given positions (values range from 0 to 100), for each of the three datasets and for each of the eight chemicals (Supplementary File S6). Note that multiple amino acids can be found as predictive features for the same amino acid position, chemical and dataset. For example, T, P and K at position 1472 were found to be predictive for response to methyl salicylate, 119-36-8, for the *D. melanogaster* model, with importance of 31.5, 30.1 and 14.4, respectively, whereas at the same position and dataset, L, F and A were predictive for response to geranyl acetate, 105-87-3, with importance of 41.0, 27.3 and 26.8, respectively.

We found that some positions were identified as predictive more often than others, which we took as evidence for their involvement in the molecular function of the OR family in general. Table 3 lists the 10 most frequently found positions in each dataset. These were selected from those that had an importance > 10 and an AUC > 0.7 (a total of 475, 457 and 151 for the *D. melanogaster*, *A. gambiae* and *H. saltator* datasets, respectively).

The top 10 predictive positions were mapped to the only available 3D structure for an insect OR (Figure 4C; PDB:6C70) [30], the Orco protein from the parasitic fig wasp *Apocrypta bakeri* (UniProtKB:B0FAQ4), using the sequence of the Orco protein from *D. melanogaster* (UniProtKB:Q9VNB5) as a link between the alignment of all ORs and the 3D structure. The ion channel structure is a heteromer of a specific OR with the OR co-receptor Orco [45,46], which opens upon ligand binding. Examination of the contact surface in the Orco tetrameric structure from *A. bakeri* suggests that the contact interface between subunits is in the lower-right part of the protein as displayed in Figure 4C. Although a patch of positions overlaps the region of subunit interaction, most are in the top domain in the region corresponding to the ligand-binding pocket (in blue in Figure 4C; mapped from [49,50,51]).

A representation of the amino acids present at each of the positions in the clusters is provided as Supplementary File S7. The correspondence between the position in the alignment and those in Orco from *A. bakeri* is indicated in Table 3 (all positions mapped in Supplementary File S8). Examination of the amino acid distributions indicates that these positions have very different behaviors regarding amino acid type and variability. For example, position 334 is mostly W (F in Orco); the *A. bakeri* Orco position is 25, in the transmembrane part of the protein. Position 2493 is mostly F, but also significantly Y and L; this is 394 in *A. bakeri* Orco, placed in the transmembrane domain and pointing outside the structure, it could be accessible for phosphorylation, and could indicate a regulatory mechanism. In contrast, other positions have much more variability, such as position 1069 or position 1472 (commented above for its association to methyl salicylate and geranyl acetate) corresponding to *A. bakeri* Orco positions 139 and 203, respectively, situated near positions equivalent to experimentally verified OR residues (see Figure 4C).

### 3.4. Relative Conservation of Predictive Residues

**Figure 5:**
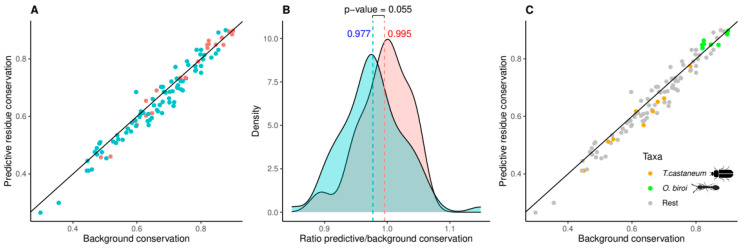
Relative conservation of predictive residues is significantly higher in clusters expanded in social insects. (**A**) Average values of conservation (predictive residues versus the background of the entire sequence) are shown for each cluster. The diagonal indicates clusters where the predictive residues are as conserved as the background. Clusters highly expanded in social insects in red; rest in blue. (**B**) Distributions of the values for ratio of predictive to background residue conservation. The 101 clusters with five or more ORs were considered for this analysis (Supplementary File S4). Clusters highly expanded in social insects (ratio ORs to species above 3.5 and more than 94% ORs from social insects; 21 clusters; red) have significantly higher relative conservation of predictive residues than other clusters (blue; average values 0.995 and 0.977, respectively; *p*-value = 0.055, Wilcoxon test). The thresholds used to segregate the clusters were based on the median values of the respective variable: (i) percentage of ORs from social insects and (ii) ratio ORs to species, respectively. (**C**) Conservation values for clusters specifically expanded in the beetle *T. castaneum* and in the ant *O. biroi* (orange and green, respectively). The clusters of the ant are mostly above the diagonal and the clusters of the beetle are mostly below the diagonal; these groups differ significantly in their ratios of residue conservation versus background (*p*-value = 0.004, Wilcoxon test).

Next, we wanted to investigate the differential conservation of residues involved in molecular function within each cluster in relation to the overall OR sequence conservation (including regulatory motifs and positions for interaction with other proteins). Therefore, we annotated each cluster with more than five ORs (101 clusters) with the ratio between amino acid conservation at the predictive positions (defined as the union of those among the top 10 of the three models; 29 residues, Table 3) and the background amino acid conservation of the entire sequence (see Section 2 for details; Supplementary File S4). We predict that clusters with high values of this ratio (i.e., having a ligand-binding pocket that is more conserved than the background) would recognize a smaller number of different odorants, while clusters with lower values of this ratio (i.e., having a binding pocket that is less conserved than the background) would recognize a broader collection of odorants. The latter could indicate evolutionary adaptation of an OR group with a conserved biological function (e.g., foraging) to different odorants (e.g., related to changes in diet). While there is a good linear correlation between predictive residue conservation and background sequence conservation (Figure 5A), their ratios range from 0.848 for C0 (one of the two large clusters that includes sequences from all 21 species) to 1.149 for C61 (containing nine ORs in three species of Neoptera) with a median value of 0.979 (Supplementary File S4). The ant-specific C2, representing largely the 9-exon family, has a value of 0.990.

The numerous expansions of OR families within social insects were considered to reflect selection pressure to improve the ability of these species to communicate chemically [6,7]. We wondered whether these OR radiations are accompanied by a narrowing or broadening of the odor tuning, i.e., whether new ORs formed by duplication in a cluster are likely to bind to very similar or very different ligands. Evidence for this would be greater or lesser relative conservation of residues predisposed to binding.

To test this hypothesis, we divided the 101 clusters in two ways: (i) we defined clusters rich in social insect ORs as those with more social insect ORs than the median of all clusters (94%; 50 clusters), and (ii) we defined highly expanded clusters as those with a ratio of ORs to species represented in the cluster above the median (3.5 ORs per species; 48 clusters). Splitting the clusters according to condition (i) or (ii) did not result in significantly different distributions of the relative conservation of predictive residuals (*p*-values of 0.313 and 0.529, respectively, Wilcoxon test). Remarkably, we observed a result closer to significance when both conditions were applied together (21 clusters; *p*-value = 0.055; Figure 5B), suggesting that clusters with many expansions in social insects indeed show a trend towards higher relative conservation of these residuals. Social evolution in insects is thus characterized by duplications of genes leading to large OR subfamilies specialized in the recognition of very similar odorants.

Focusing on the two insects from our set of 21 species with the highest number of species-specific extended clusters, the flour beetle *T. castaneum* (nine clusters in Figure 5C; orange), a non-social insect, and the clonal raider ant *O. biroi* (eight clusters in Figure 5C; green), a social insect, we find that the OR clusters specifically expanded in the beetle have a lower level of residue conservation versus background than those specifically expanded in the ant (0.956 and 1.014, respectively; *p*-value = 0.004). These figures suggest that the evolutionary and functional processes associated with the OR family must differ between these species. Our observations indicate that the observed extensions of OR families in different orders may be regarded as adaptations to chemical environments with different odor spectra. The expansion of the OR repertoire of the beetle *T. castaneum* allows perception of a wide diversity of different odorants, whereas in the clonal raider ant a similar number of OR family expansions provided detection of a narrower range of chemically similar odorants.

## 4. Discussion

In this work, we have presented a new approach that can help to extend the functional characterization of the large family of OR proteins through the annotation and analysis of a large amount of sequence data. Our approach starts with the collection and curation of selected datasets of insect ORs. The alignment of 3902 protein sequences provides a framework for comparing functional information from these sequences. Positions in this alignment were mapped to a template structure available for an ancestral protein of the family [30].

Using machine learning, we examined three separate, functionally characterized datasets, and for each of them we predicted sets of residues responsible for ligand recognition. While most of the predictive positions correspond to the region of the ligand binding pocket, the presence of some positions in the region of subunit interaction suggests that we could be detecting other types of functional residues related to interactions of the protein and not directly to the binding of the ligand (Figure 4C). Independently, we used a sequence-based clustering algorithm to divide the family into clusters expected to be responsible for related functions in the same or different organisms. Finally, we annotated these clusters with respect to their taxonomic distribution, identifying clusters with particular expansion patterns and with different relative conservation of residues predicted to be responsible for ligand recognition. Our results suggest that the large expansions of the OR family in social insects are associated with subfamilies that recognize very similar ligands (Figure 5). Expansions leading to subfamilies with broader recognition ranges may be more common in non-social species, such as the flour beetle *T. castaneum*.

Our work facilitates the analysis of the ORs of 21 insect species in terms of the information we have obtained for the whole family. These data are available through a dedicated web service called iOrME. Potentially, additional individual ORs of species not included in our set of 21 species can be added by including them in the multiple sequence alignment of 3902 sequences. In this sense, all mapped information can flow from and to new OR sequences of interest.

We are aware that the results presented in our work are inevitably influenced by species selection, which itself reflects a bias in this area of research, but we have attempted to remove such biases by defining variables that can be applied to different taxonomic levels and that are normalized by values, such as the number of species or the conservation of whole sequences. As part of our efforts to remove these biases, we plan to add new OR datasets as needed in order to expand our coverage of OR functionality, and in principle it should be easy to include new experimental data and information from new protein structures as they become available. Our dedicated website is a resource that will accommodate newer versions of the OR dataset, clusters, machine learning results and annotations.

The OR family is not the only large protein family with large paralogous expansions (see, e.g., ubiquitination-related families in Chlamydiae [52], or the families of F-box proteins in plants [53]). We propose that an approach similar to the one we have presented here could be similarly applied to other expanded families, irrespective of their function or taxonomic distribution. We expect that from the study of many such families, we will obtain further insights into the rules that drive gene duplication and gain of function.

## Figures and Tables

**Table 1:** List of insect species used and number of raw and curated Odorant Receptors.

Species	Tax ID	Taxonomy (Order > Suborder > Family)	Raw Number OR	Curated Number OR
*Calopteryx splendens*	52612	Odonata > Zygoptera > Calopterygidae	5	5
*Cloeon dipterum*	197152	Ephemeroptera > Pisciforma > Baetidae	50	24
*Blattella germanica*	6973	Blattodea > - > Ectobiidae	135	89
*Aphis glycines*	307491	Hemiptera > Sternorrhyncha > Aphididae	47	42
*Acyrthosiphon pisum*	7029	Hemiptera > Sternorrhyncha > Aphididae	87	67
*Pediculus humanus*	121224	Phthiraptera > Anoplura > Pediculidae	13	10
*Tribolium castaneum*	7070	Coleoptera > Polyphaga > Tenebrionidae	338	253
*Manduca sexta*	7130	Lepidoptera > Glossata > Sphingidae	74	50
*Anopheles gambiae*	7165	Diptera > Nematocera > Culicidae	79	73
*Drosophila melanogaster*	7227	Diptera > Brachycera > Drosophilidae	61	61
*Nasonia vitripennis*	7425	Hymenoptera > Apocrita > Pteromalidae	211	199
*Bombus terrestris*	30195	Hymenoptera > Apocrita > Apidae	165	149
*Apis mellifera*	7460	Hymenoptera > Apocrita > Apidae	160	153
*Harpegnathos saltator*	610380	Hymenoptera > Apocrita > Formicidae	377	360
*Ooceraea biroi*	2015173	Hymenoptera > Apocrita > Formicidae	574	501
*Linepithema humile*	83485	Hymenoptera > Apocrita > Formicidae	367	323
*Camponotus floridanus*	104421	Hymenoptera > Apocrita > Formicidae	407	376
*Solenopsis invicta*	13686	Hymenoptera > Apocrita > Formicidae	396	287
*Pogonomyrmex barbatus*	144034	Hymenoptera > Apocrita > Formicidae	293	293
*Acromyrmex echinatior*	103372	Hymenoptera > Apocrita > Formicidae	435	306
*Atta cephalotes*	12957	Hymenoptera > Apocrita > Formicidae	434	281

**Table 2:** Keys for taxonomic labels.

Code	Taxon
(A)	Insecta
(B)	Palaeoptera
(B1)	*C. splendens*
(B2)	*C. dipterum*
(C)	Neoptera
(D)	*B. germanica*
(E)	Paraneoptera
(F)	Hemiptera
(F1)	*A. glycines*
(F2)	*A. pisum*
(G)	*P. humanus*
(H)	Endopterygota
(I)	*T. castaneum*
(J)	*M. sexta*
(K)	Diptera
(K1)	*A. gambiae*
(K2)	*D. melanogaster*
(L)	Hymenoptera
(M)	*N. vitripennis*
(N)	Aculeata
(O)	Apoidea
(O1)	*B. terrestris*
(O2)	*A. mellifera*
(P)	Formicoidea
(Q)	*H. saltator*
(R)	Formicoids
(S)	*O. biroi*
(T)	Formicoids − *O. biroi*
(U)	*L. humile*
(V)	Myrmicinae + *C. floridanus*
(W)	*C. floridanus*
(X)	Myrmicinae
(X1)	*S. invicta*
(X2)	*P. barbatus*
(Y)	Attini
(Y1)	*A. echinatior*
(Y2)	*A. cephalotes*

**Table 3:** Top predictive positions in the multiple sequence alignment as obtained from the machine learning approach per dataset. We mapped the top predictive positions from the multiple sequence alignment to the sequence of the ORCO_DROME protein (UniProtKB:Q9VNB5), and to its homologous protein B0FAQ4_APOBA (UniProtKB:B0FAQ4), for which there is an available 3D structure (PDB:6C70). Datasets: 1 = *D. melanogaster*, 2 = *A. gambiae*, 3 = *H. saltator*.

Dataset	Times Predictive	Alignment Position	*D. melanogaster* Orco Position	*A. bakeri* Orco Position	Amino Acid (D.m./A.b.)
1	8	1472	207	203	L/V
1	7	508	67	63	N/E
1	7	430	48	44	V/V
1	7	2529	414	402	R/R
1	7	2493	406	394	F/F
1	7	1069	143	139	T/T
1	7	2581	420	408	S/S
1	7	2855	486	474	K/K
1	7	620	83	79	F/F
1	7	1398	197	193	I/F
2	14	1210	170	166	S/E
2	11	1208	168	164	T/T
2	10	2345	387	375	V/V
2	10	430	48	44	V/V
2	9	1024	-	-	-/-
2	9	550	70	66	E/D
2	9	2591	421	409	S/S
2	9	2391	392	380	F/A
2	9	1594	229	225	E/E
2	9	334	30	25	F/F
3	2	216	16	11	D/D
3	2	1380	-	-	-/-
3	2	865	110	106	Q/N
3	2	1607	232	228	Q/Q
3	2	2771	-	-	-/-
3	2	2600	424	412	E/E
3	2	601	77	73	N/N
3	2	993	-	-	-/-
3	2	1474	208	204	F/I
3	2	2827	479	467	F/F

## Data Availability

The datasets used, produced, and analyzed in our study are available both as Supplementary Data and in the iOrME repository (http://cbdm-01.zdv.uni-mainz.de/~munoz/iorme/; accessed on 21 April 2022).

## Supplementary Materials

The following supporting information can be downloaded at: https://www.mdpi.com/article/10.3390/genes13050919/s1, Figure S1: Number of proteins in each cluster for subfamilies defined by gene models for species *A. cephalotes, A. echinatior, C. floridanus* and *H. saltator*; Figure S2: Average F1, precision and sensitivity values during 10-fold cross-validations repeated 10 times of the machine learning models (see Methods for details); File S1: Multiple sequence alignment of all 3902 OR sequences considered in the analysis; File S2: Binarized response data of OR proteins from 3 datasets to 8 chemicals. Columns indicate dataset (#1 = *D. melanogaster*, #2 = *A. gambiae*, #3 = *H. saltator*), id—cluster identifier, ac—UniProt AC, following eight columns—registry numbers of the panel of chemicals (see Methods for IUPAC and common names); File S3: Clusters of OR proteins; File S4: Annotated clusters. Columns indicate ID—cluster ID, Number OR—number of ORs, Protein length—average length with standard deviation, Number of species, Common taxonomy, Bg conservation—conservation of the entire sequence, Predictive conservation—conservation of predictive residues, Ratio predictive/bg—ratio between the conservation of predictive residues and the entire sequence, Social ratio—ratio of ORs from social insects, ORs/species—ratio between the number of ORs and the number of species in the cluster. See Methods for details; File S5: Classification performance of the machine learning models (a model for each chemical-dataset pair). Columns indicate dataset (#1 = *D. melanogaster*, #2 = *A. gambiae*, #3 = *H. saltator*), chem_id—chemical ID, mtry—number of variables in each tree of the forest (random forest parameter), auc—area under roc curve, auc_ci—auc confidence interval, sen—sensitivity, sen_ci—sensitivity confidence interval, pre—precision, pre_ci—precision confidence interval, f1—F1 performance; File S6: Importance of machine learning features (alignment position and amino acid) by model (a model for each chemical-dataset pair). Columns indicate importance, auc—area under ROC curve of the related model, dataset (#1 = *D. melanogaster*, #2 = *A. gambiae*, #3 = *H. saltator*), chem_id—chemical ID, position—position in the multiple sequence alignment, aa—amino acid; File S7: Amino acid usage for each of the predictive positions in clusters; File S8: Mapping of the positions from the OR alignment to the Orco proteins from *D. melanogaster* and *A. bakeri*.
