# Supplementary material for: Annotation and Analysis of 3902 Odorant Receptor Protein Sequences from 21 Insect Species Provide Insights into the Evolution of Odorant Receptor Gene Families in Solitary and Social Insects
Source: Genes (Basel). 2022 May 20;13(5):919. doi: 10.3390/genes13050919 (PMC9141868; doi:10.3390/genes13050919)

Cross-Validation Performance

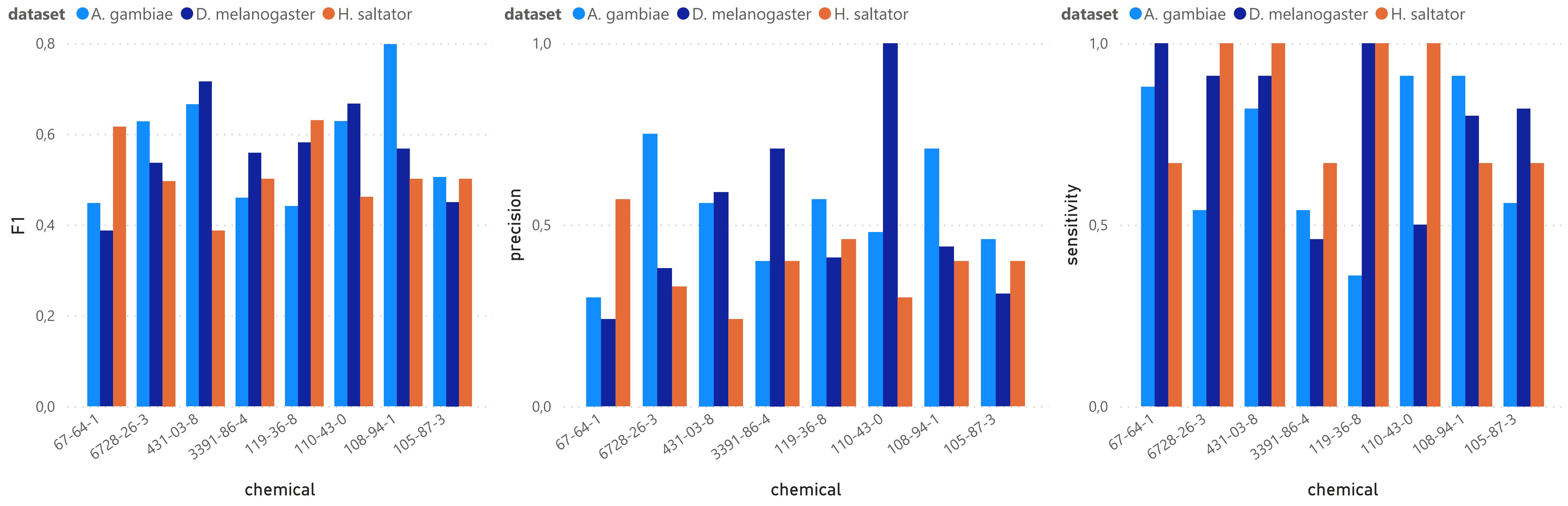

Supplement: Supplementary file 1 [file genes-13-00919-s001.zip › SupplementaryFiles_OR/Supplementary Figure S2.pdf]
