# Supplementary material for: Annotation and Analysis of 3902 Odorant Receptor Protein Sequences from 21 Insect Species Provide Insights into the Evolution of Odorant Receptor Gene Families in Solitary and Social Insects
Source: Genes (Basel). 2022 May 20;13(5):919. doi: 10.3390/genes13050919 (PMC9141868; doi:10.3390/genes13050919)
Supplement: Supplementary file 1 [file genes-13-00919-s001.zip › SupplementaryFiles_OR/Supplementary File S7.pdf]

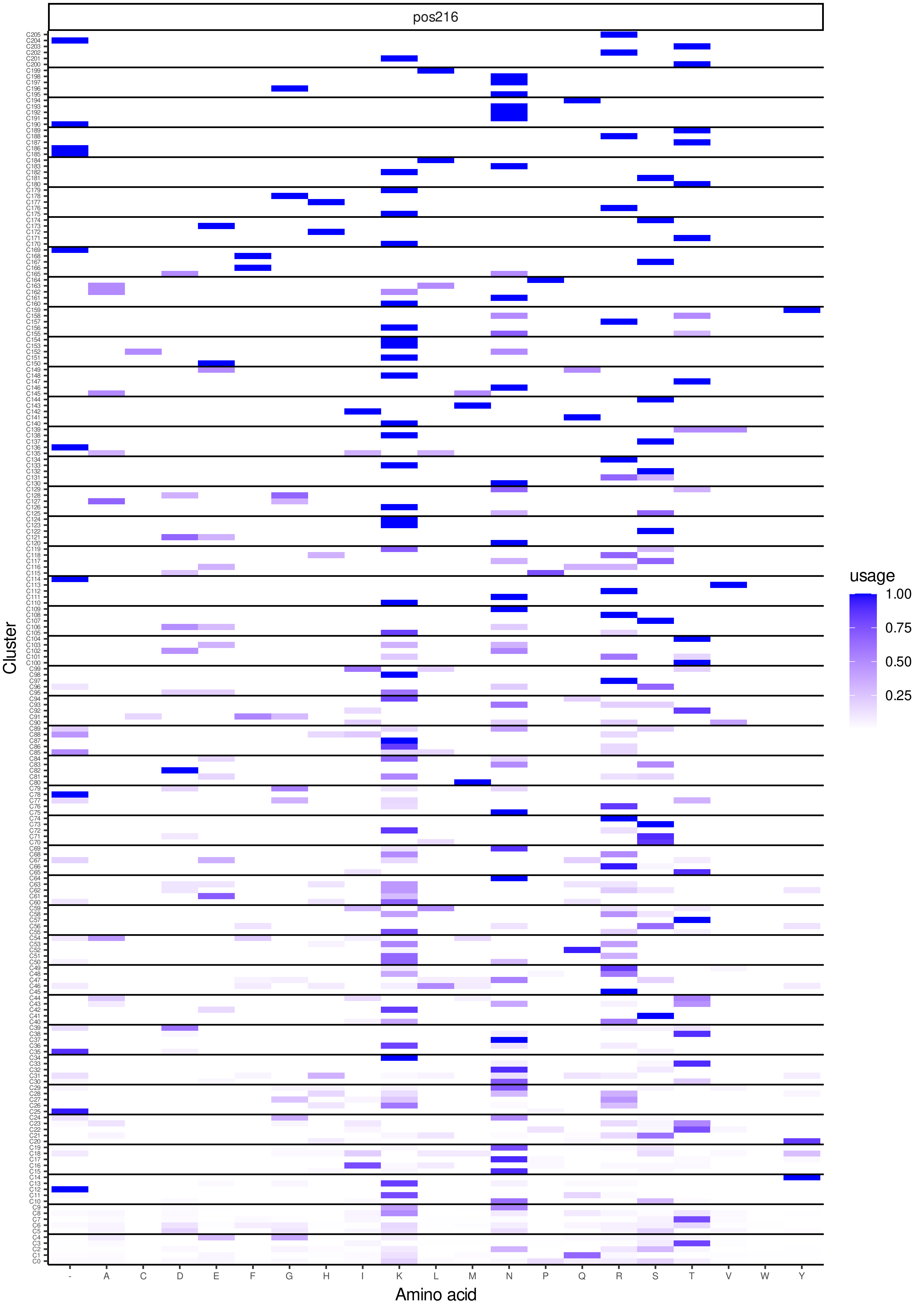
pos216
Cluster
Amino acid
- A C D E F G H I K L M N P Q R S T V W Y
usage
1.00
0.75
0.50
0.25

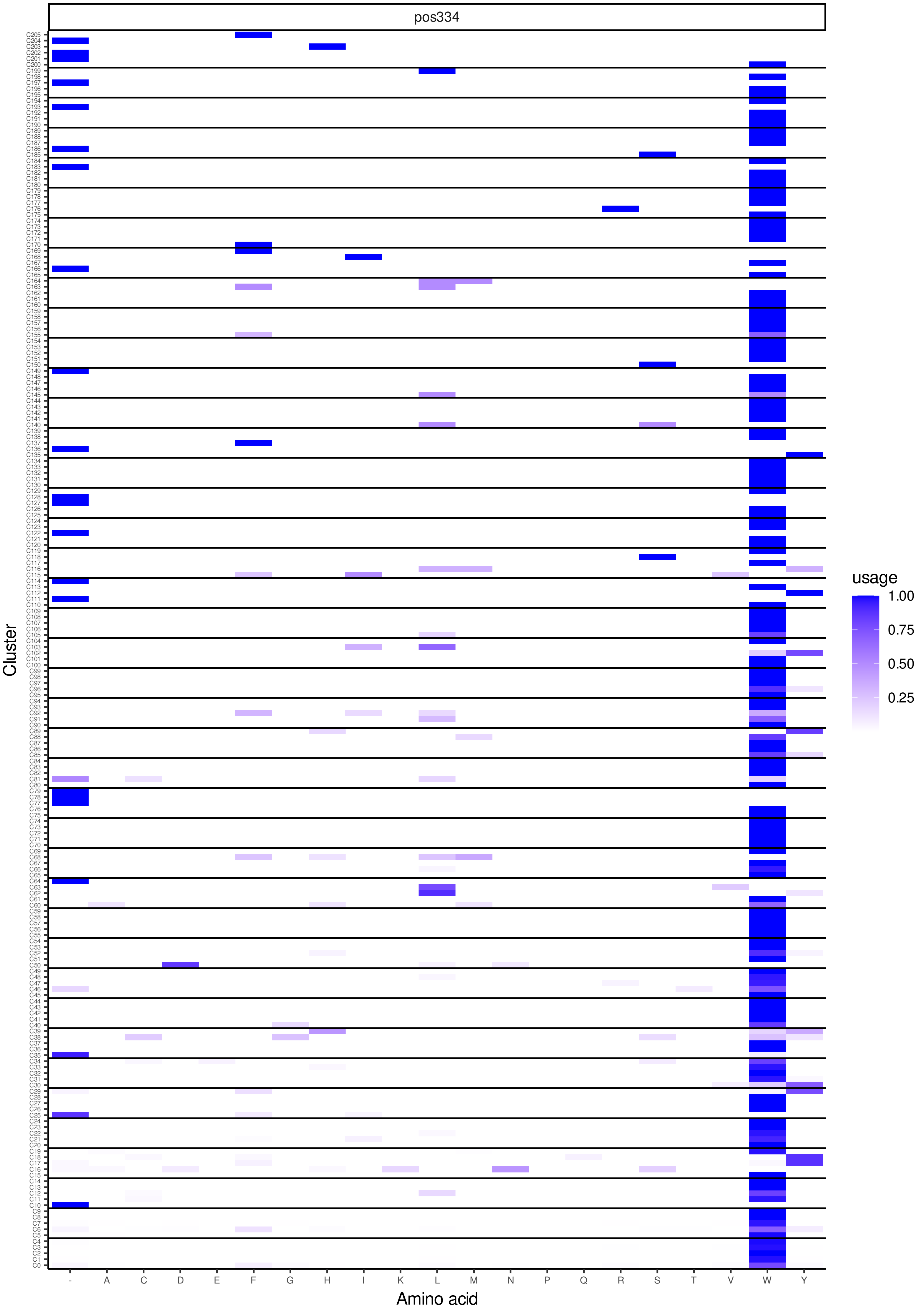
pos334
Cluster
Amino acid
- A C D E F G H I K L M N P Q R S T V W Y
usage
1.00
0.75
0.50
0.25

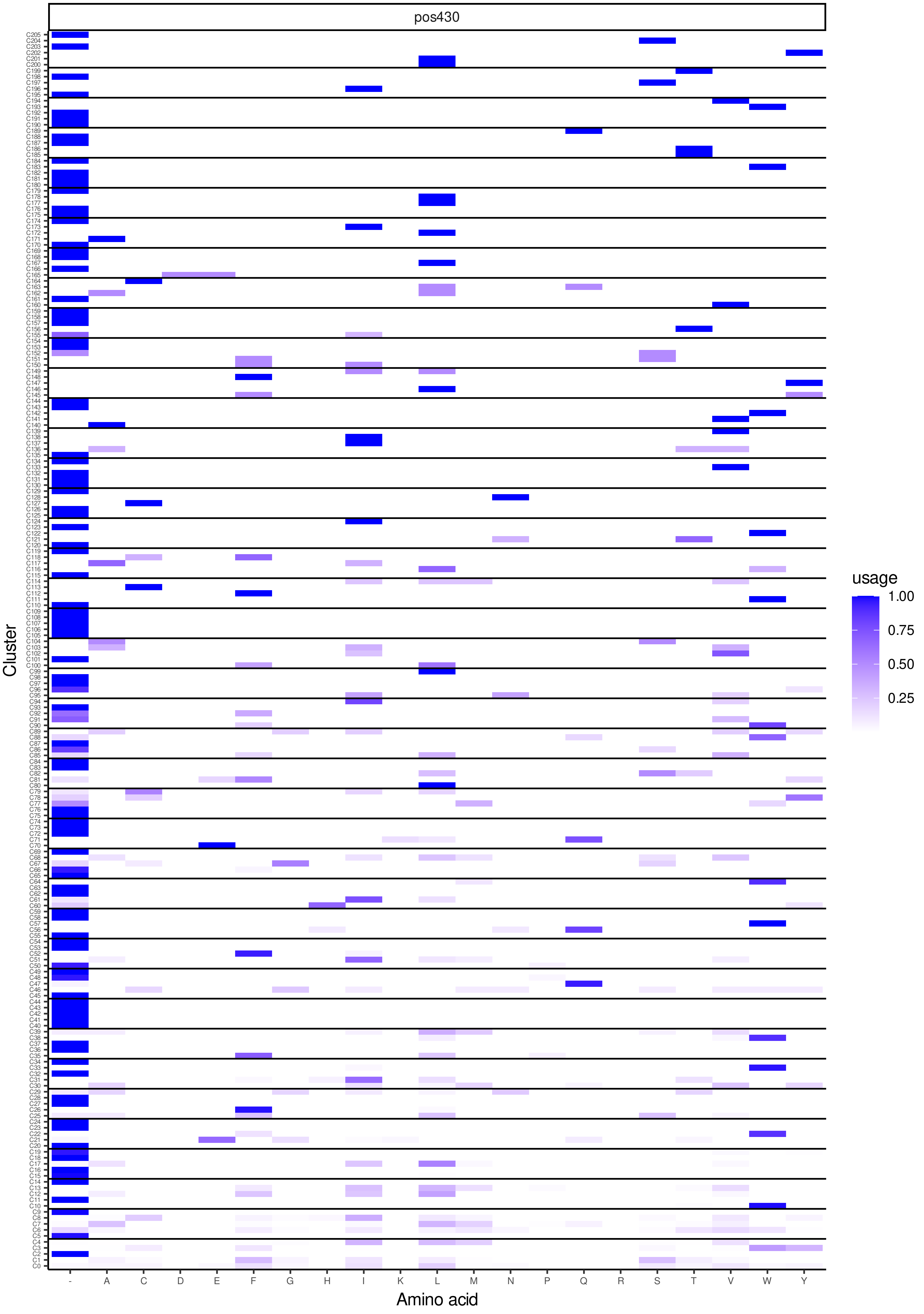
pos430
Cluster
Amino acid
usage
1.00
0.75
0.50
0.25

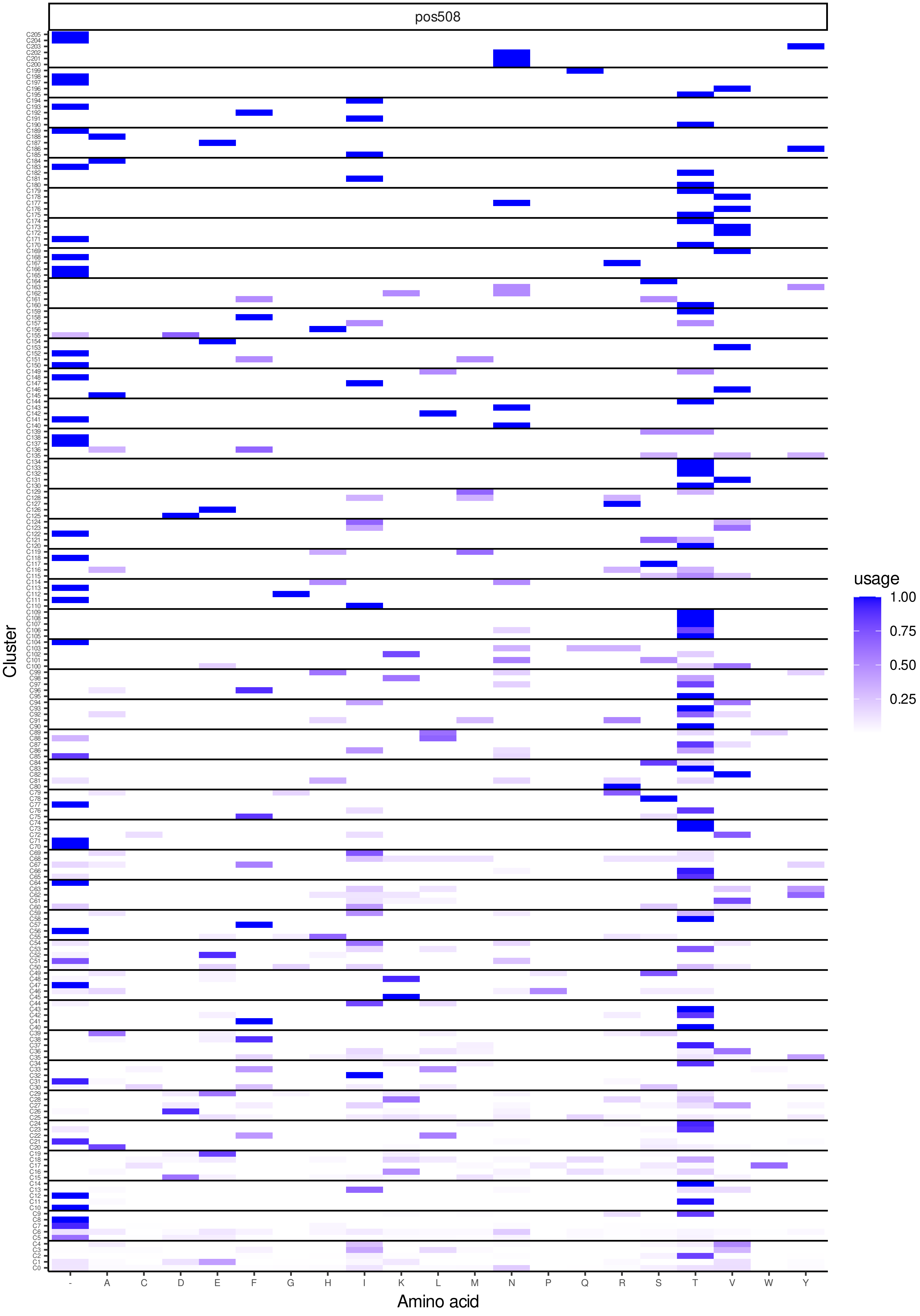
pos508
Cluster
Amino acid
usage
1.00
0.75
0.50
0.25
- A C D E F G H I K L M N P Q R S T V W Y
C0 … C205

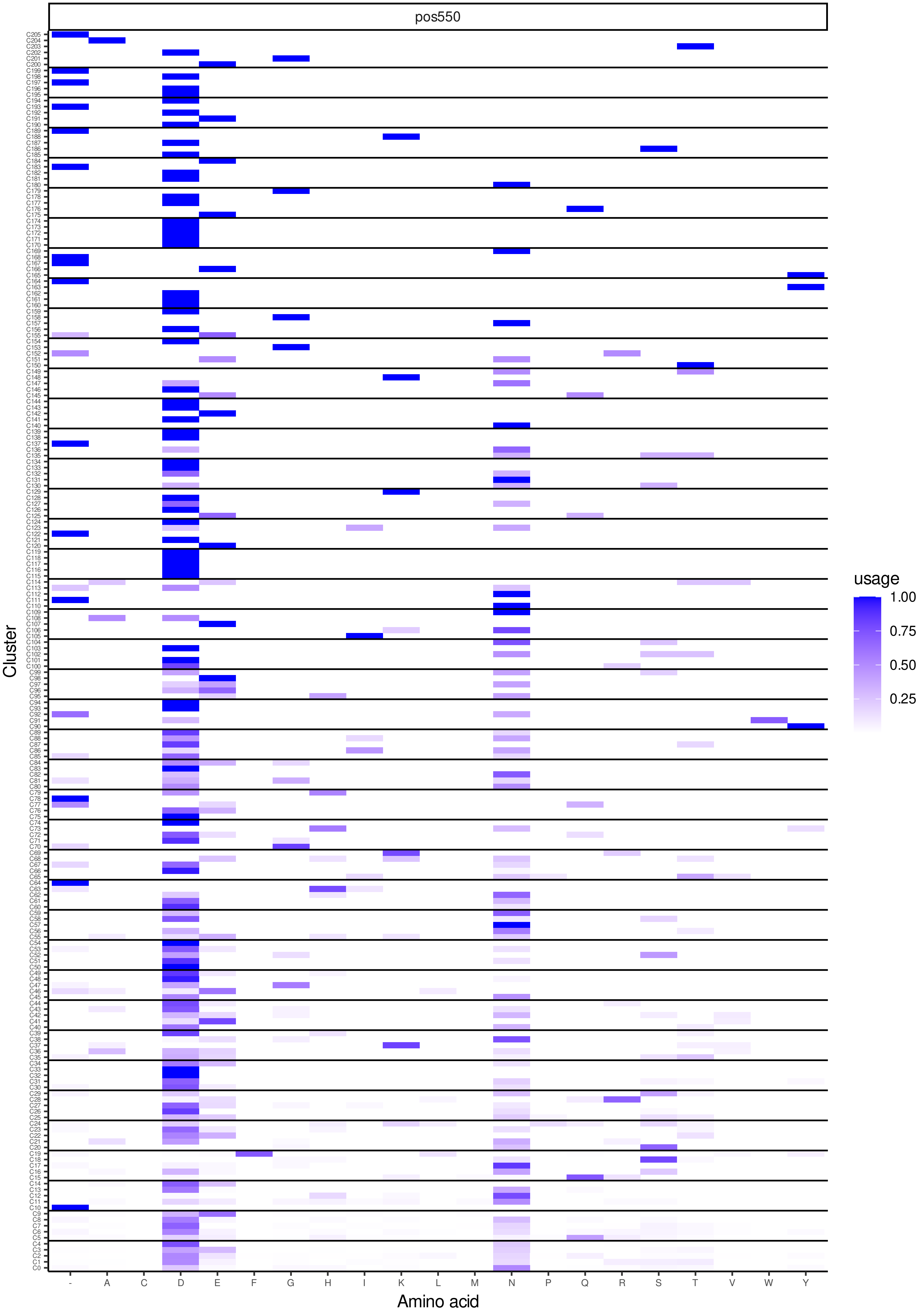

pos550
Cluster
Amino acid
- A C D E F G H I K L M N P Q R S T V W Y
usage
1.00
0.75
0.50
0.25

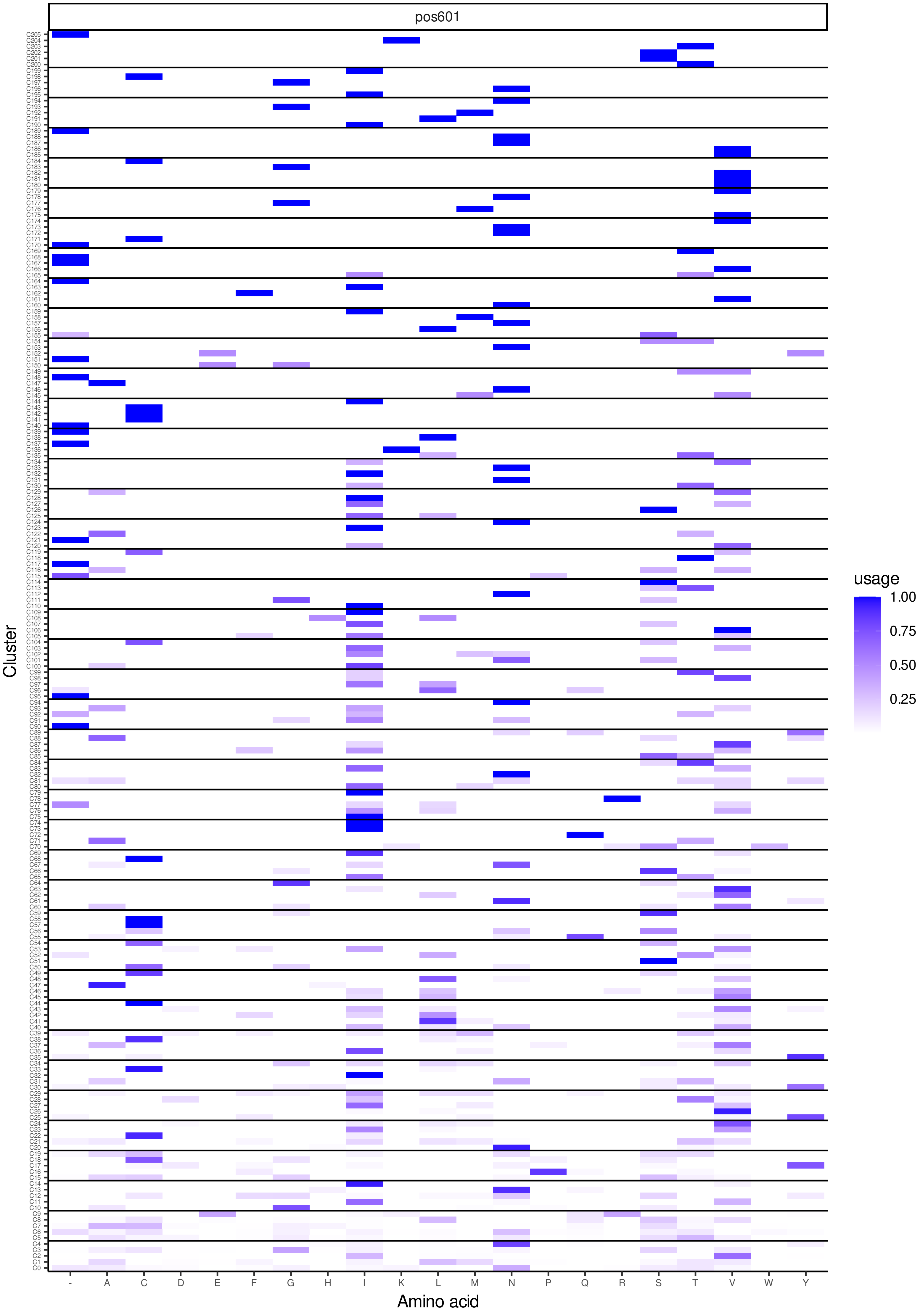
pos601
Cluster
- A C D E F G H I K L M N P Q R S T V W Y
Amino acid
usage
1.00
0.75
0.50
0.25

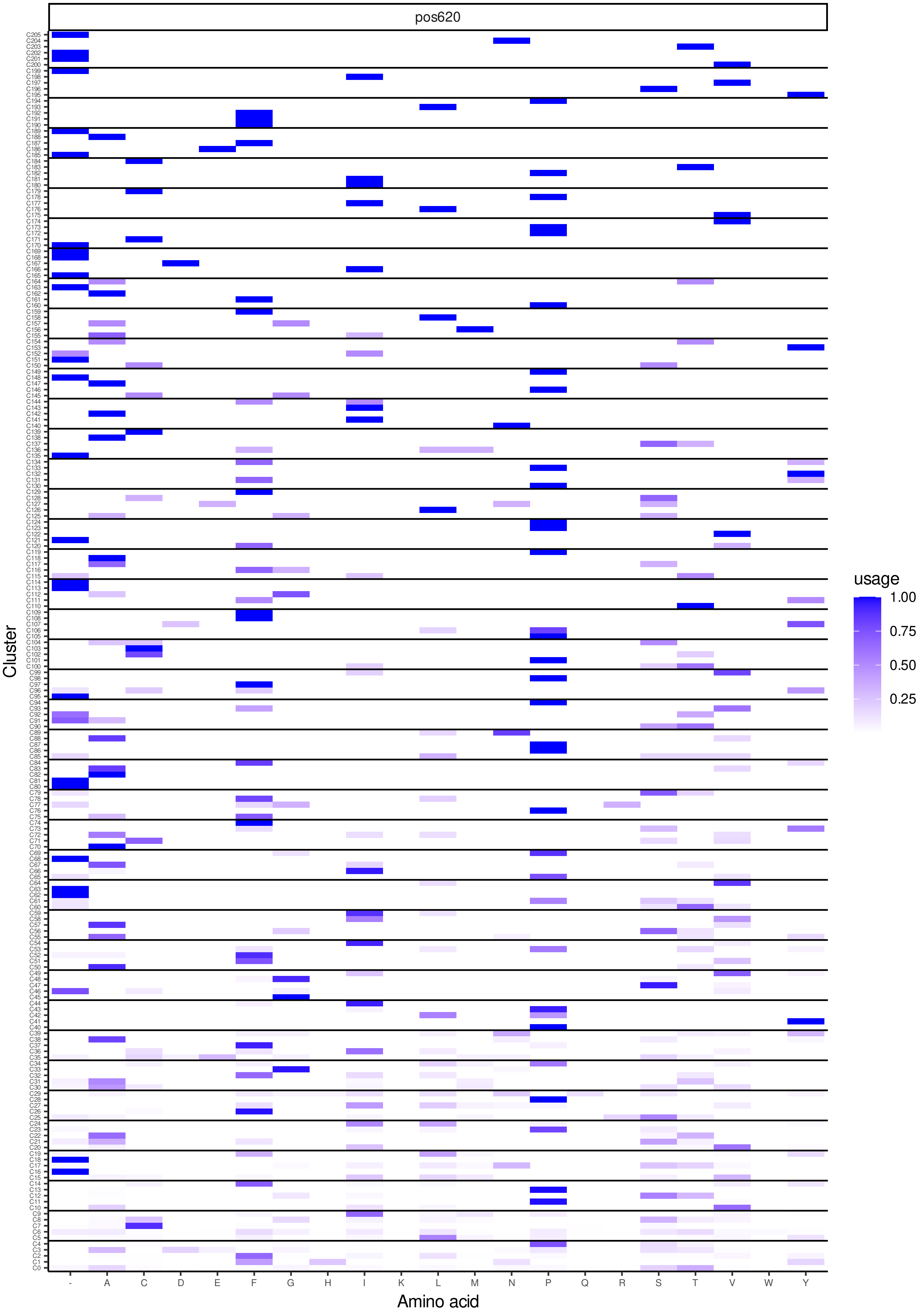

pos620
Cluster
Amino acid
- A C D E F G H I K L M N P Q R S T V W Y
usage
1.00
0.75
0.50
0.25
C205
C0

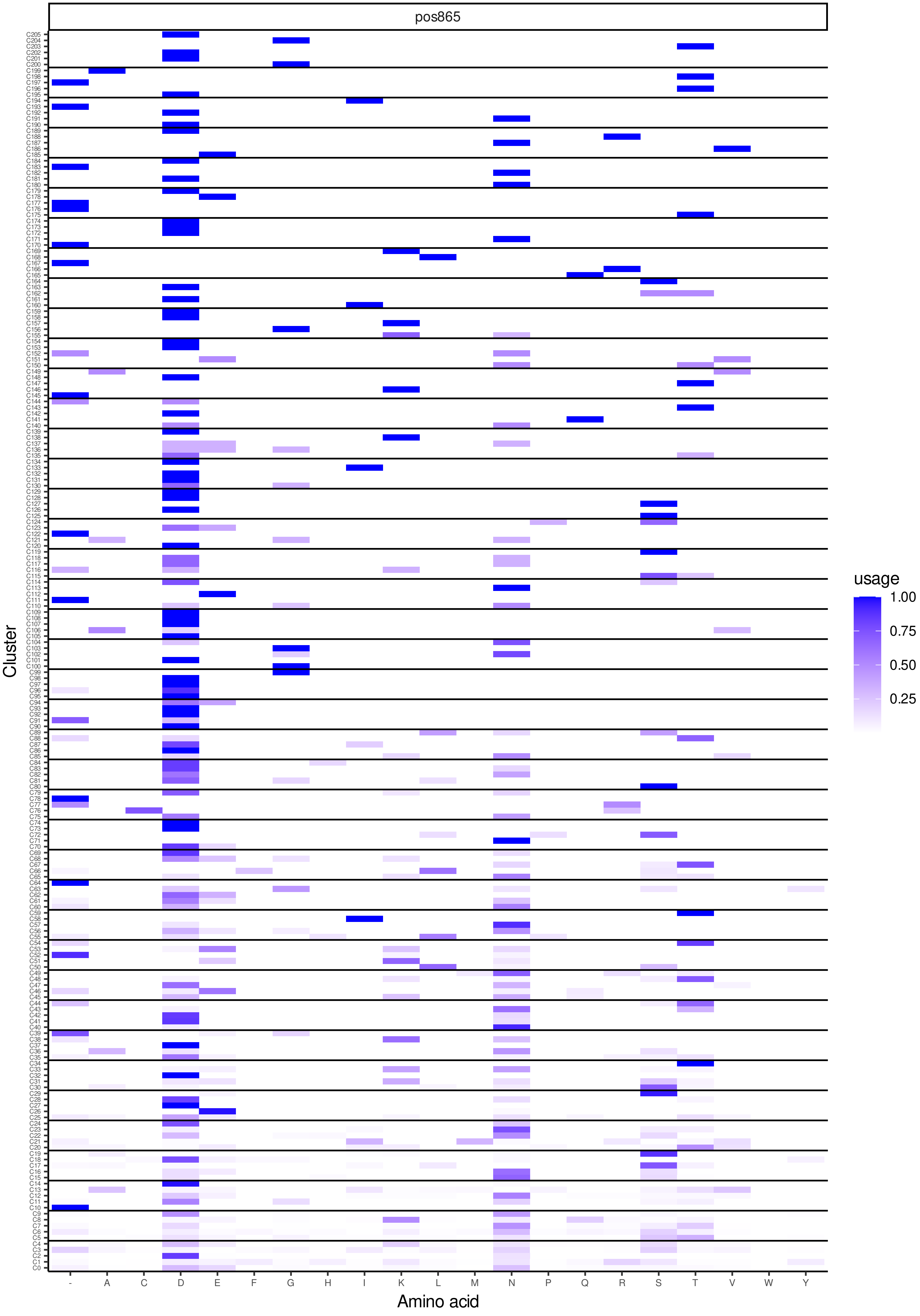

pos865
Cluster
Amino acid
- A C D E F G H I K L M N P Q R S T V W Y
usage
1.00
0.75
0.50
0.25

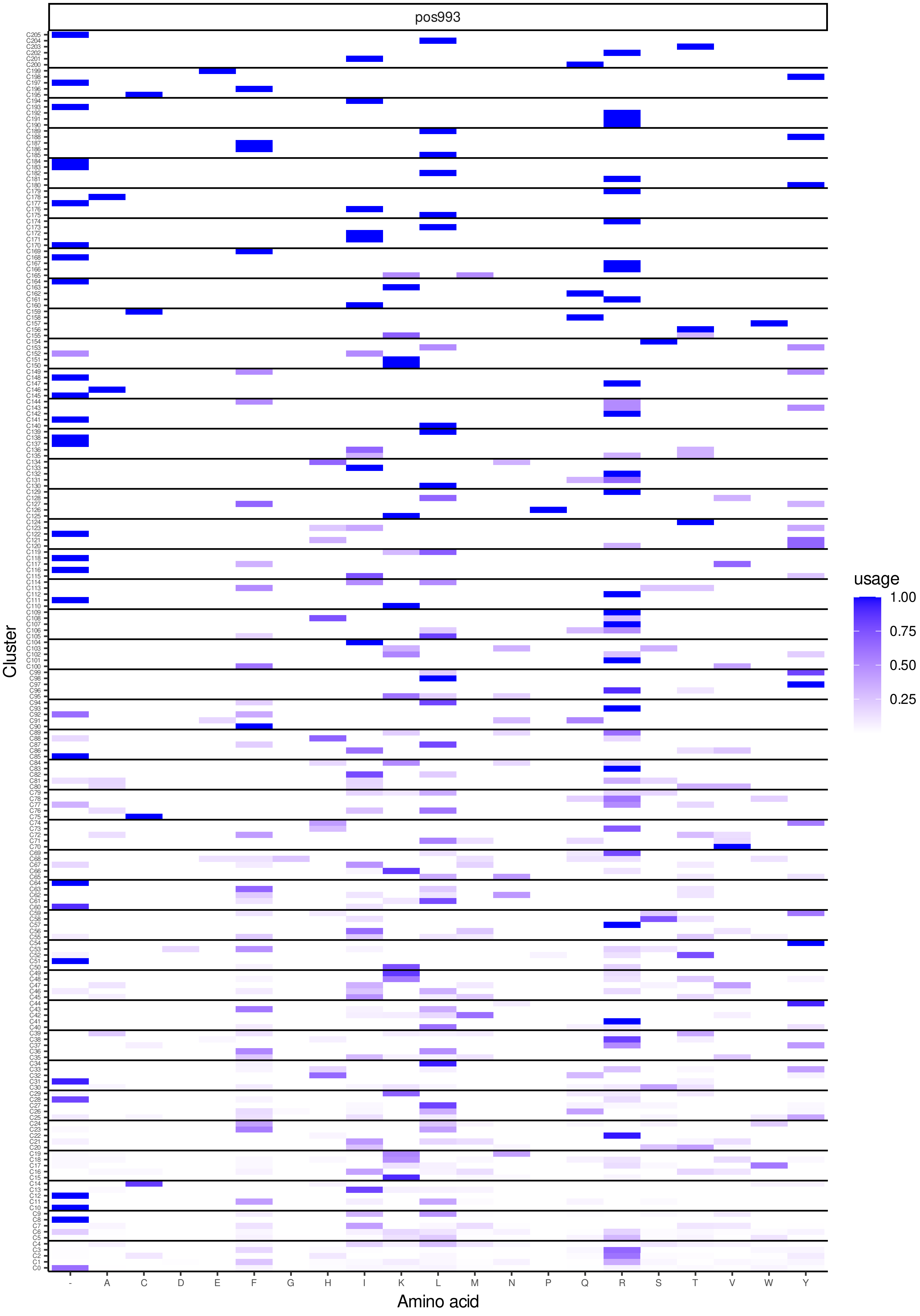

pos993
Cluster
Amino acid
usage
1.00
0.75
0.50
0.25
- A C D E F G H I K L M N P Q R S T V W Y

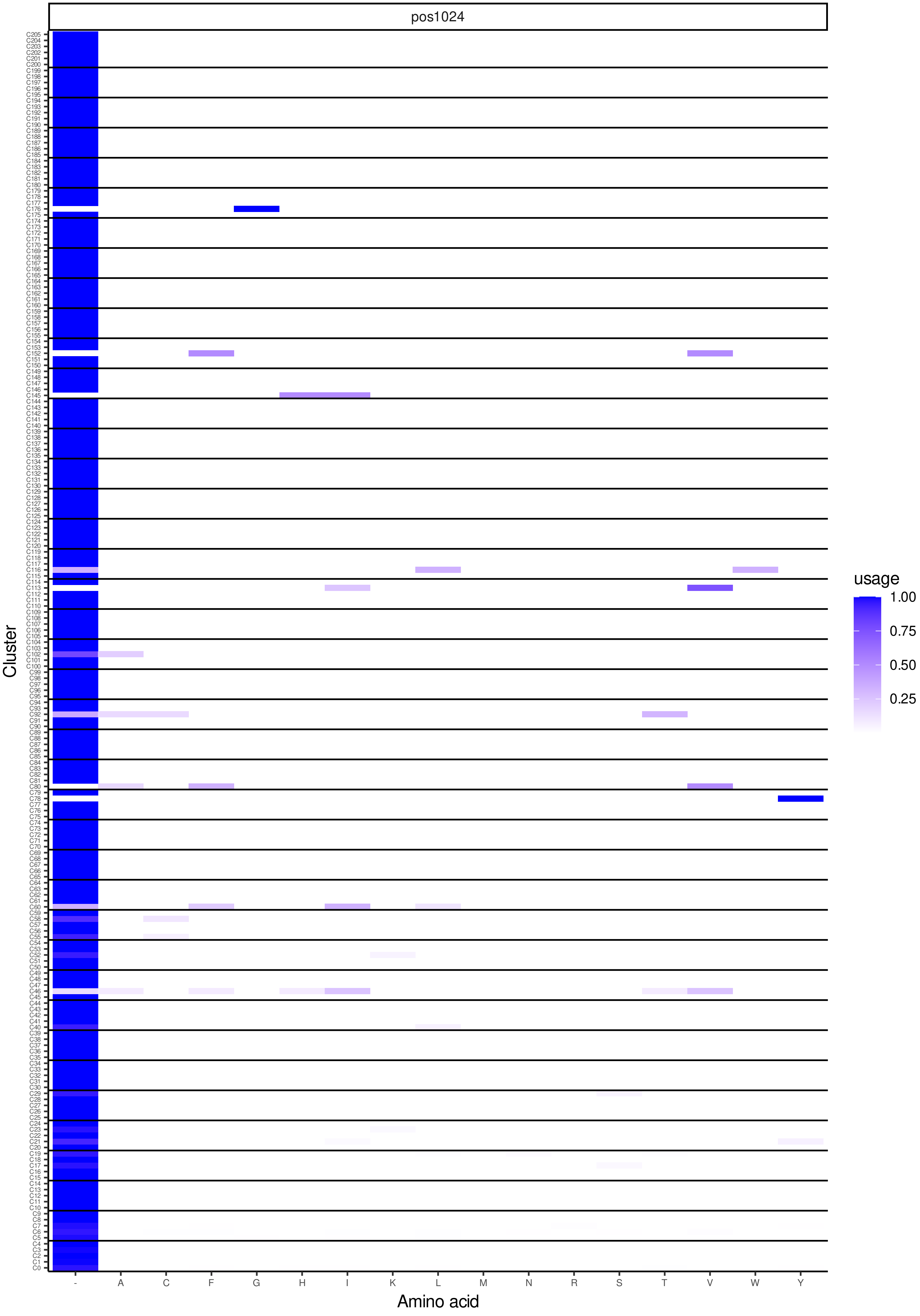
pos1024
Cluster
Amino acid
- A C F G H I K L M N R S T V W Y
usage
1.00
0.75
0.50
0.25

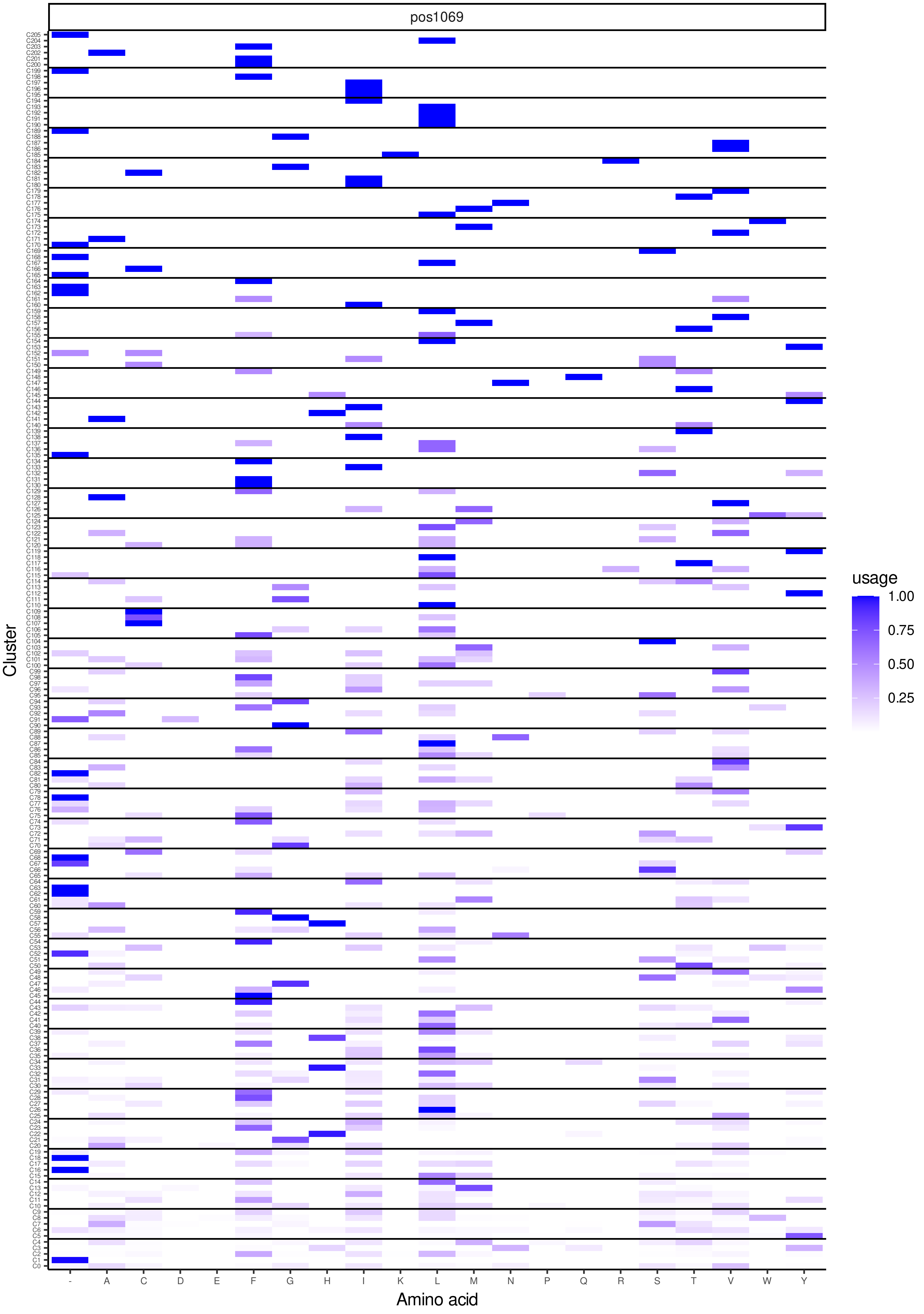
pos1069
Cluster
Amino acid
- A C D E F G H I K L M N P Q R S T V W Y
usage
1.00
0.75
0.50
0.25

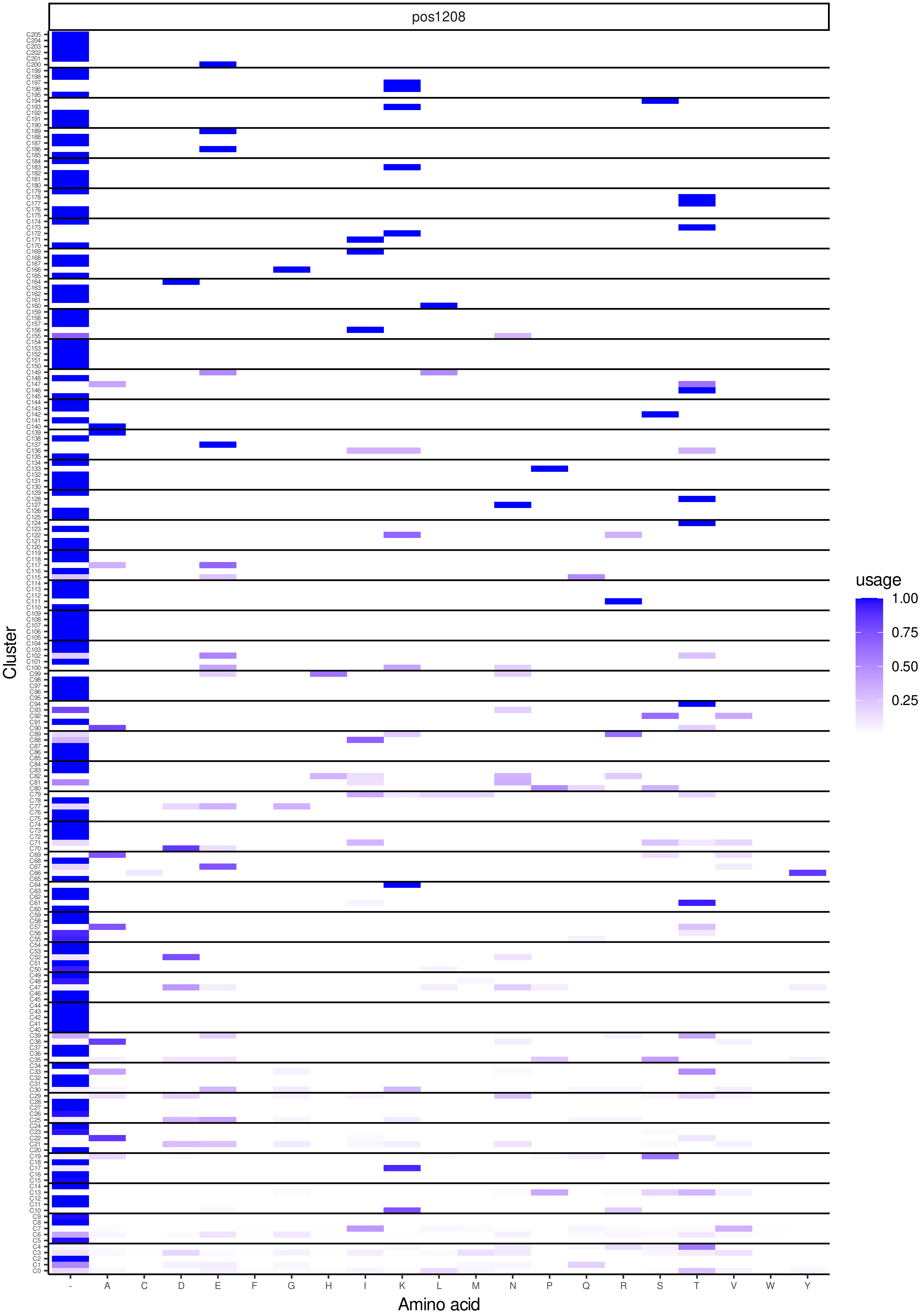
pos1208
Cluster
Amino acid
usage
1.00
0.75
0.50
0.25
- A C D E F G H I K L M N P Q R S T V W Y

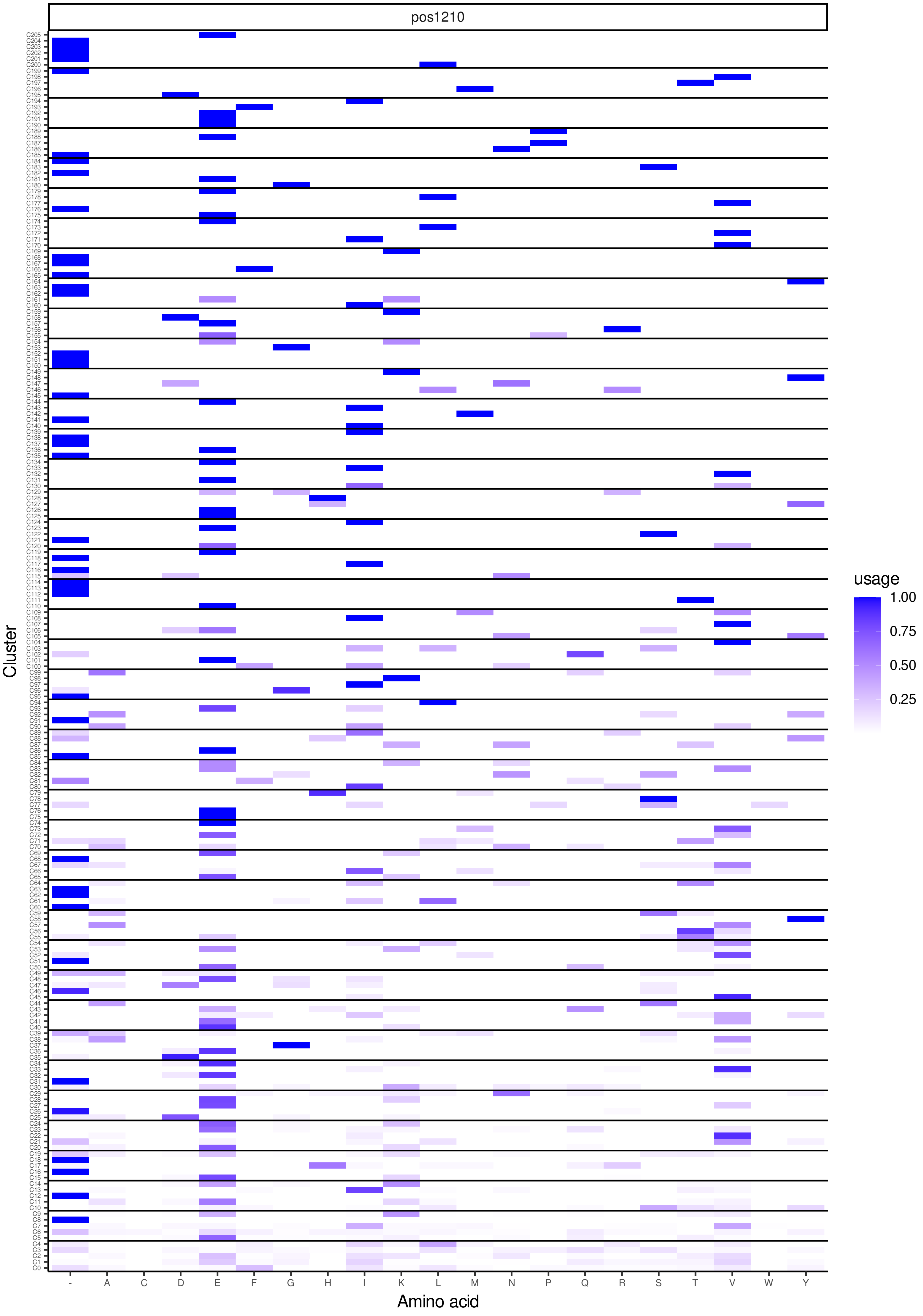
pos1210
usage
1.00
0.75
0.50
0.25
Cluster
Amino acid
- A C D E F G H I K L M N P Q R S T V W Y

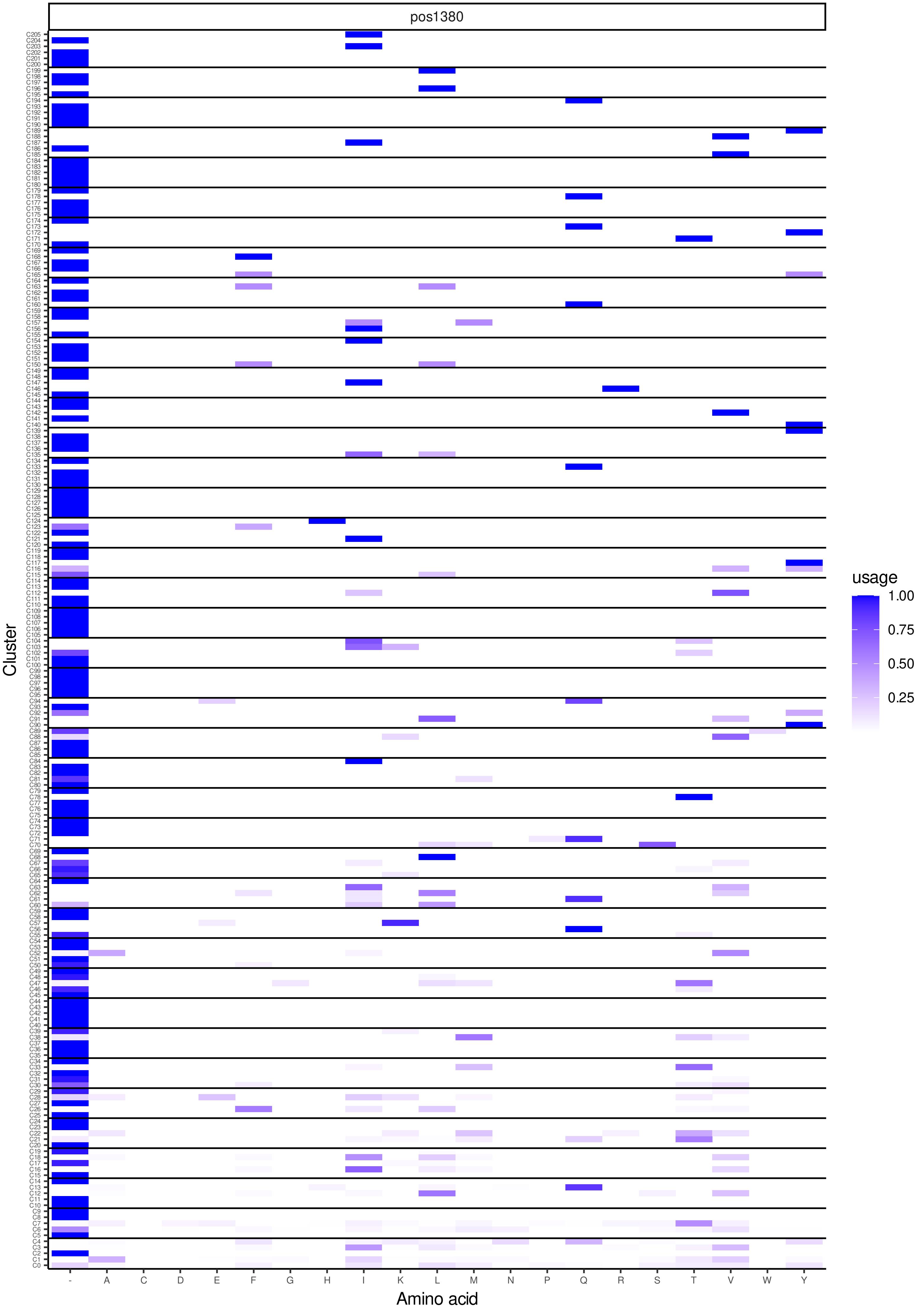

pos1380
Cluster
Amino acid
- A C D E F G H I K L M N P Q R S T V W Y
usage
1.00
0.75
0.50
0.25

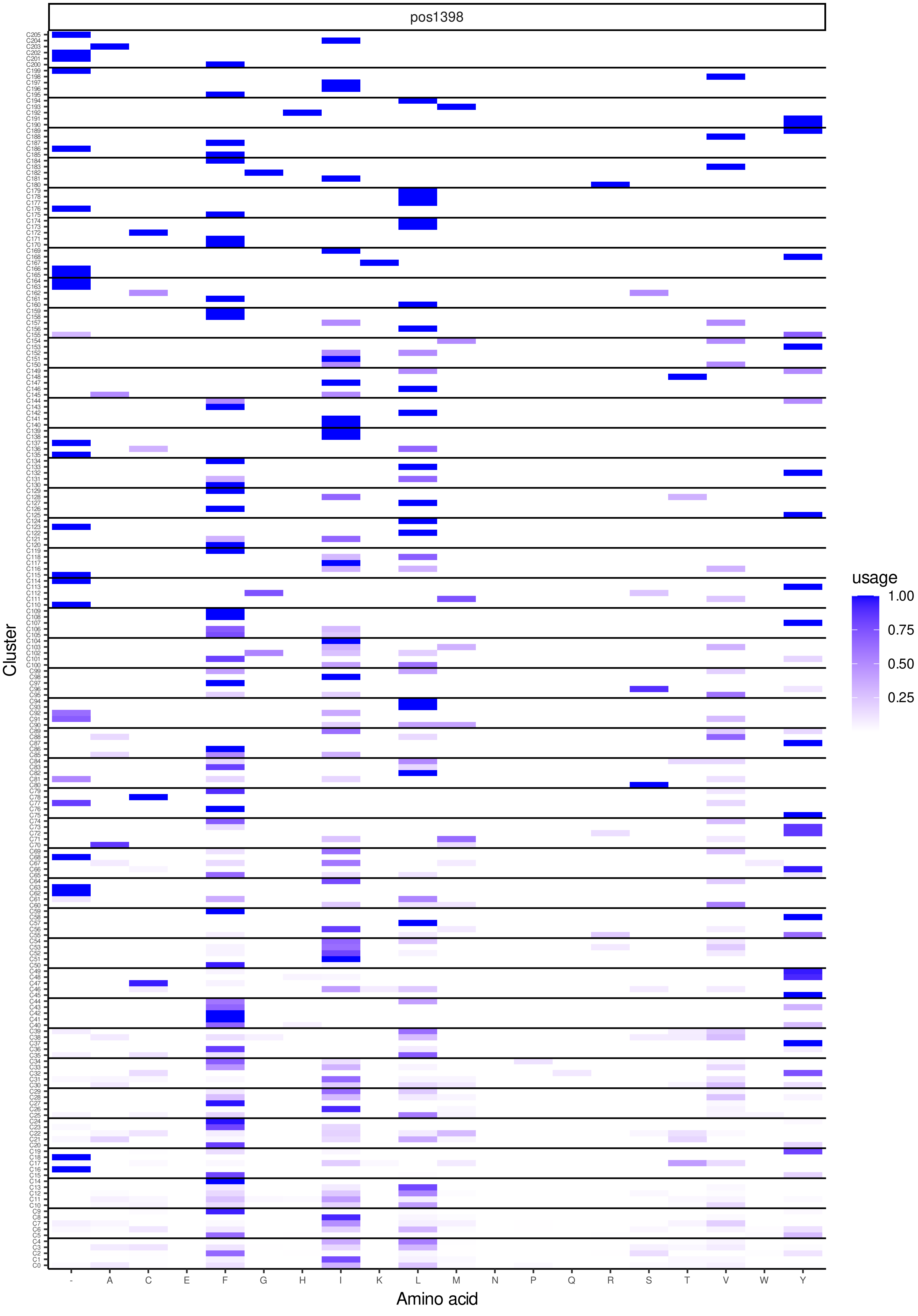
pos1398
Cluster
Amino acid
- A C E F G H I K L M N P Q R S T V W Y
usage
1.00
0.75
0.50
0.25

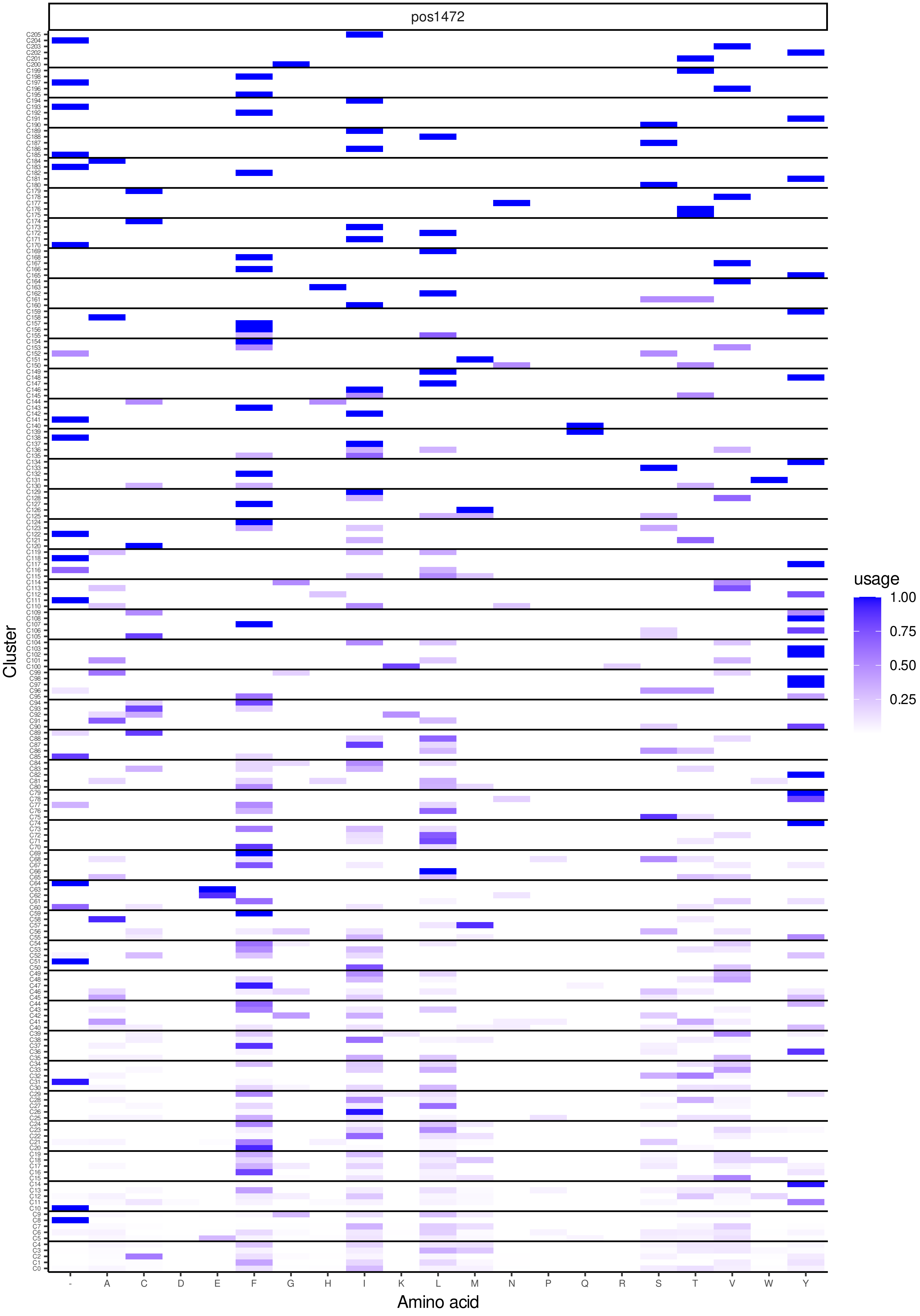
pos1472
usage
1.00
0.75
0.50
0.25
Cluster
Amino acid
- A C D E F G H I K L M N P Q R S T V W Y

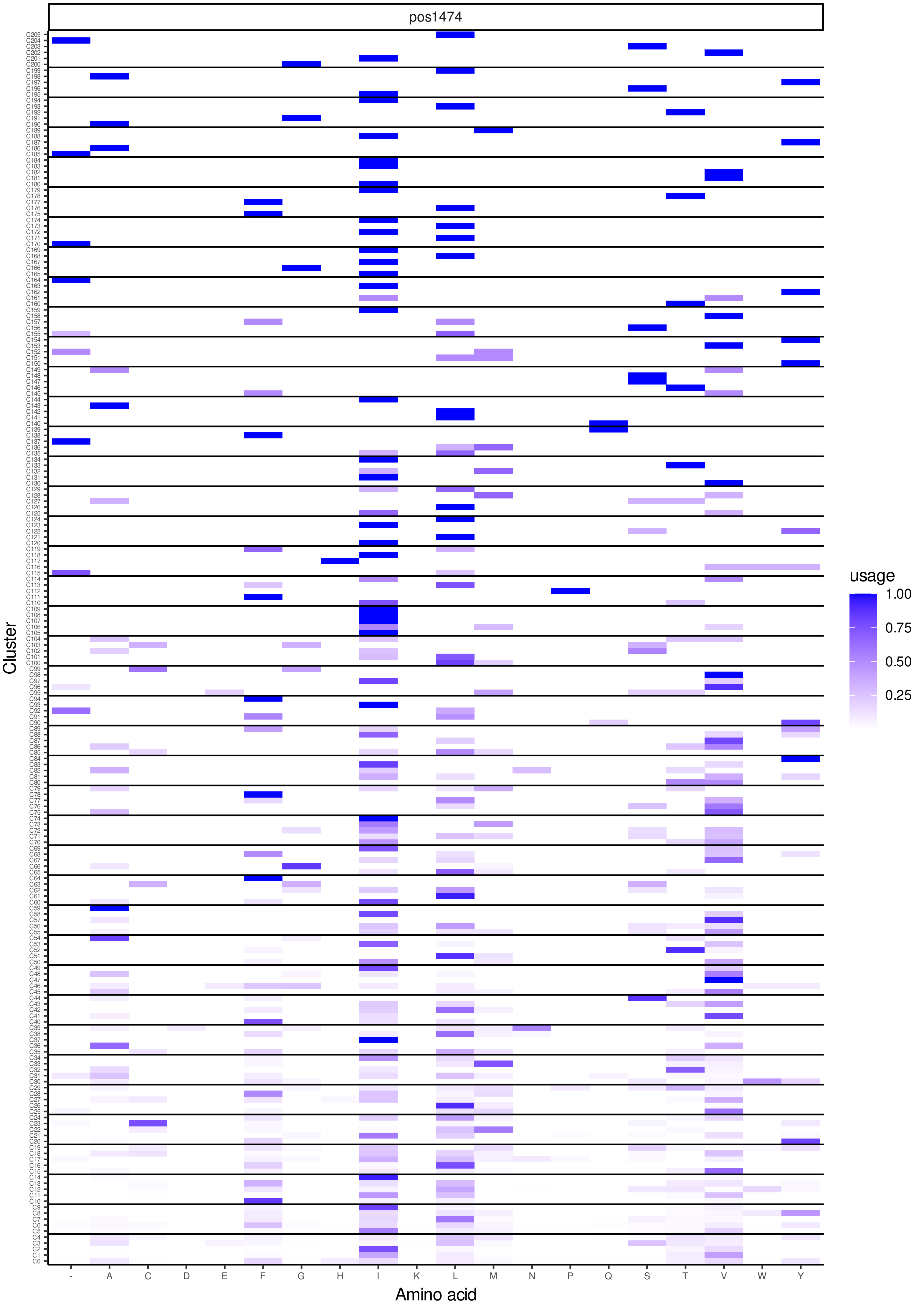
pos1474
Cluster
Amino acid
- A C D E F G H I K L M N P Q S T V W Y
usage
1.00
0.75
0.50
0.25

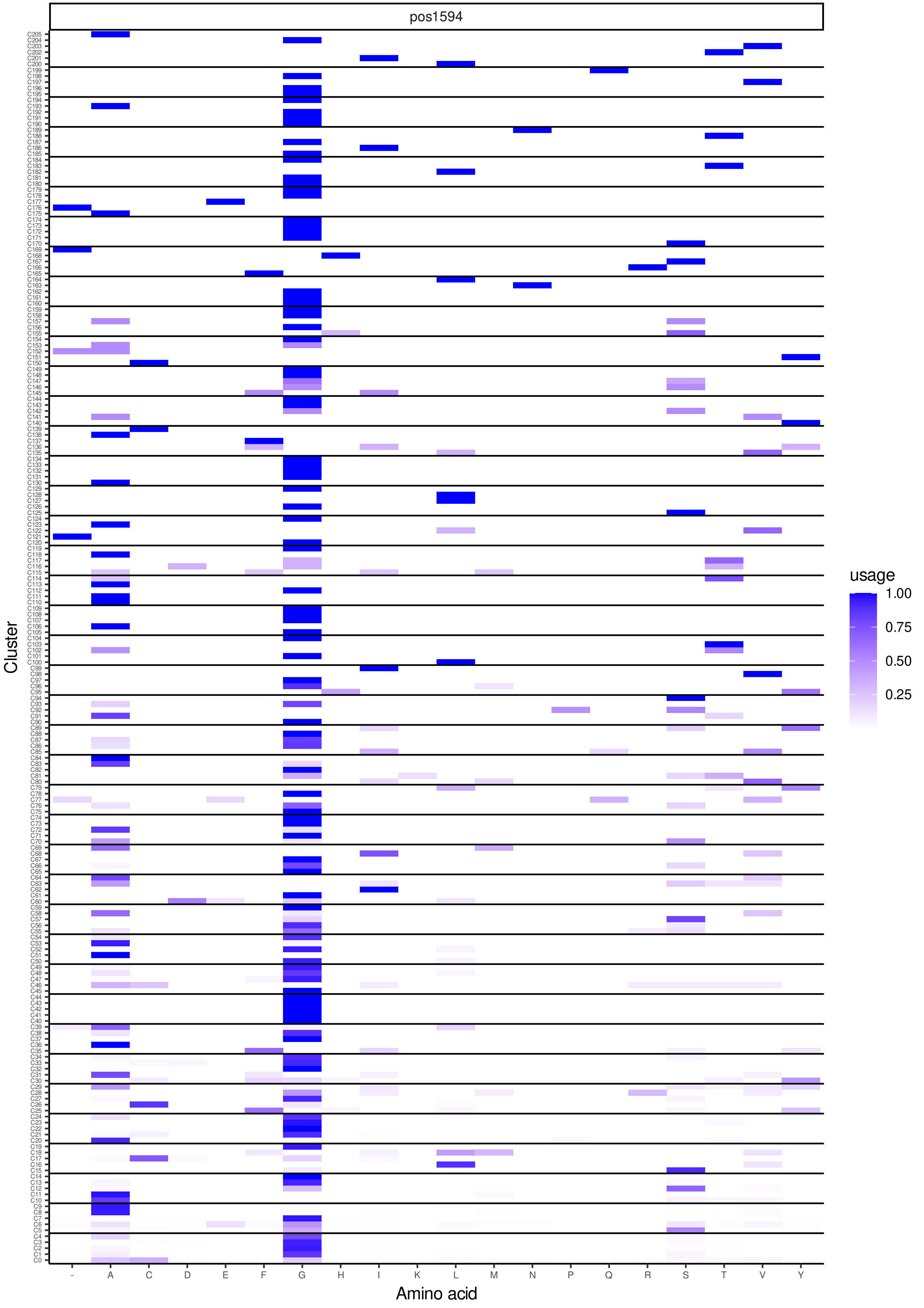
pos1594
Cluster
Amino acid
- A C D E F G H I K L M N P Q R S T V Y
usage
1.00
0.75
0.50
0.25

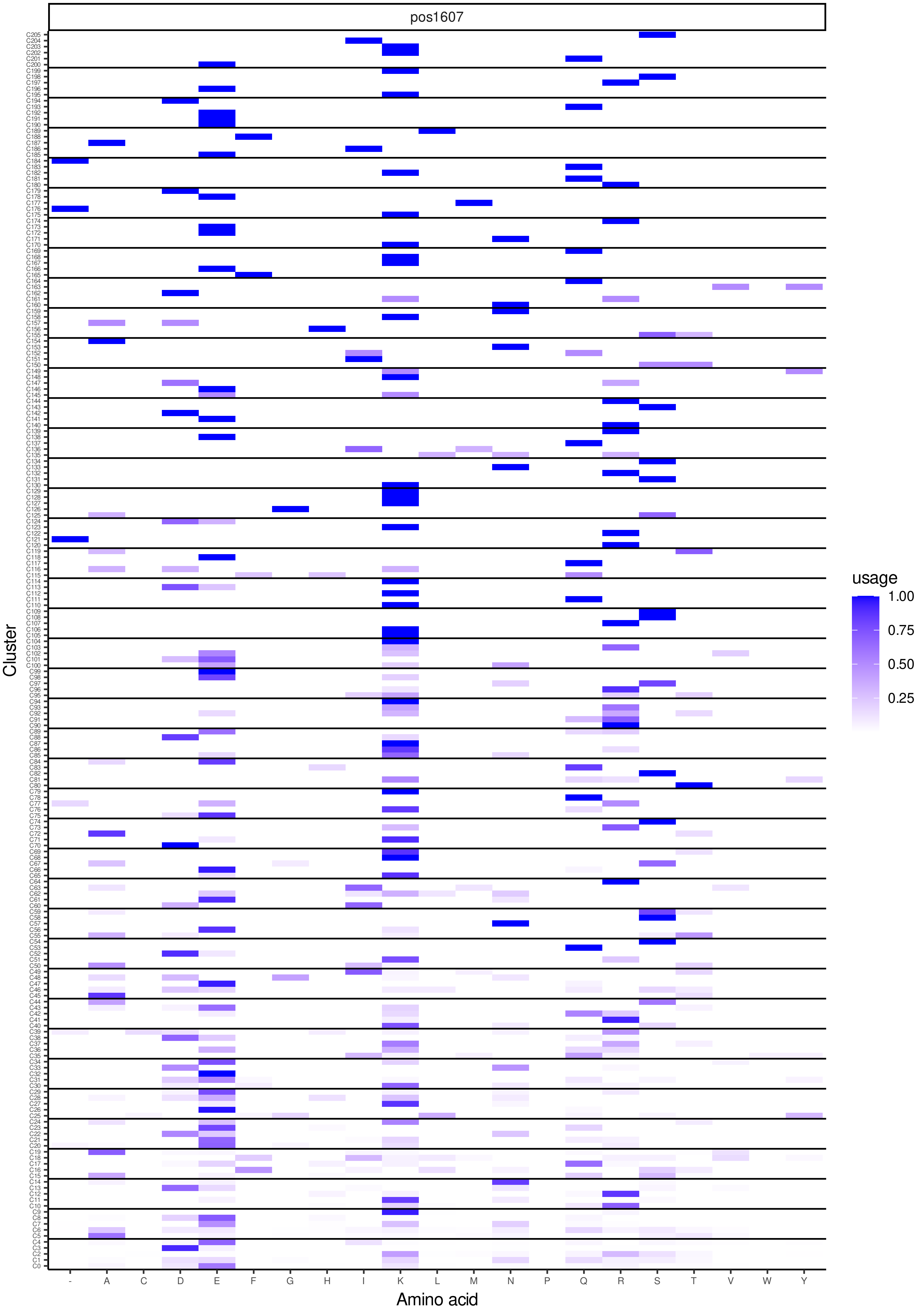
pos1607
Cluster
Amino acid
usage
1.00
0.75
0.50
0.25
- A C D E F G H I K L M N P Q R S T V W Y

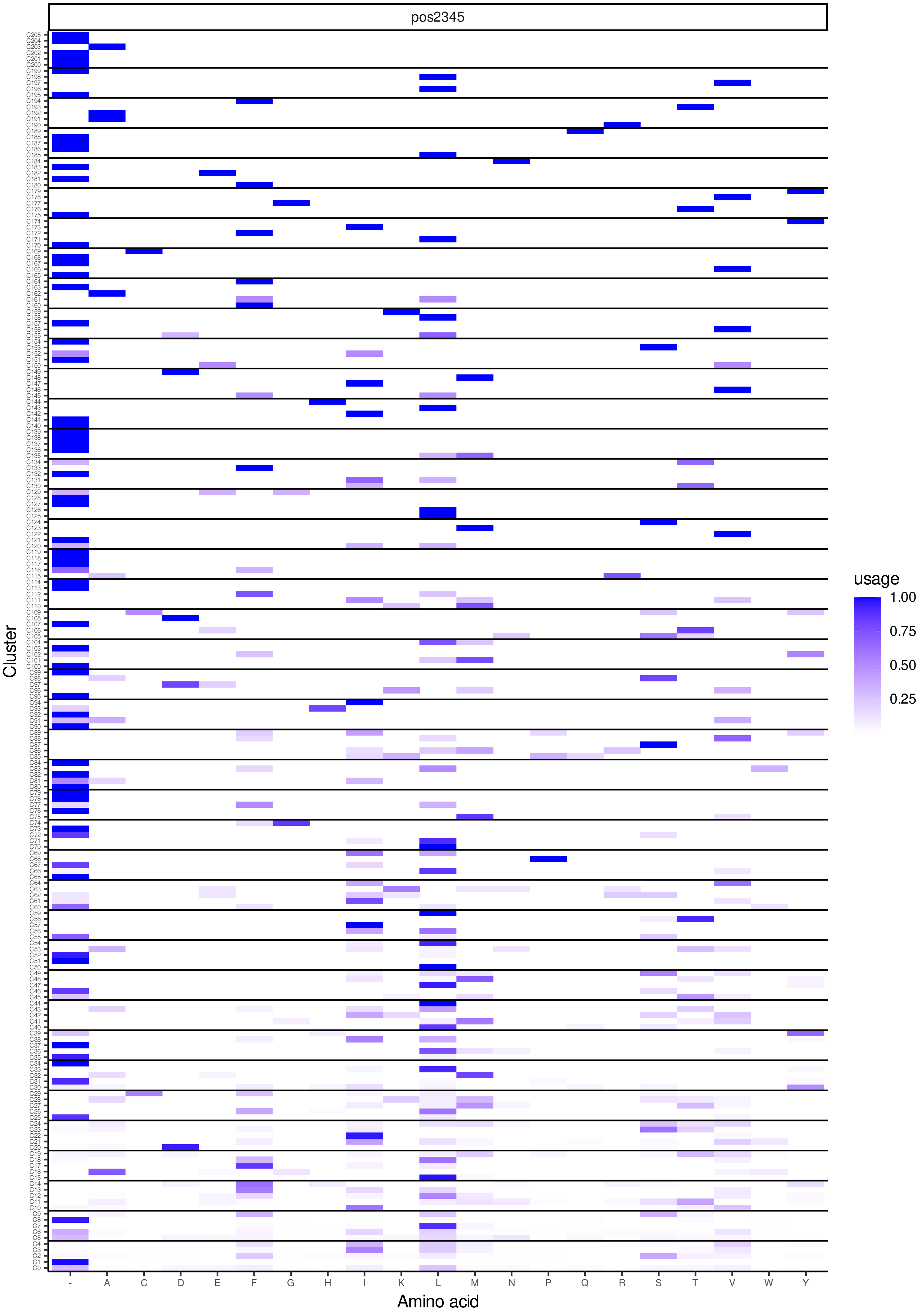
pos2345
Cluster
Amino acid
usage
1.00
0.75
0.50
0.25
- A C D E F G H I K L M N P Q R S T V W Y

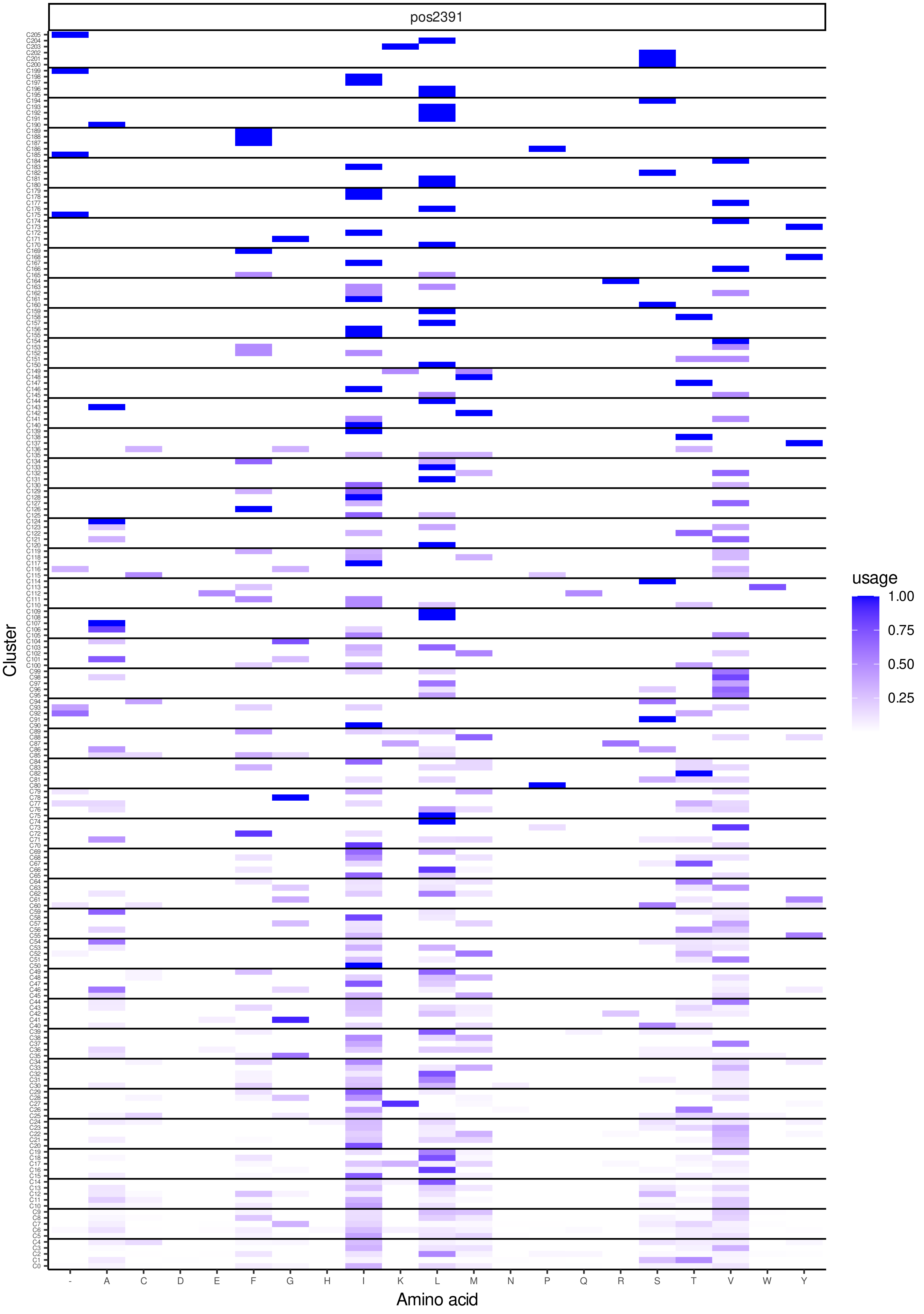
pos2391
Cluster
Amino acid
- A C D E F G H I K L M N P Q R S T V W Y
usage
1.00
0.75
0.50
0.25

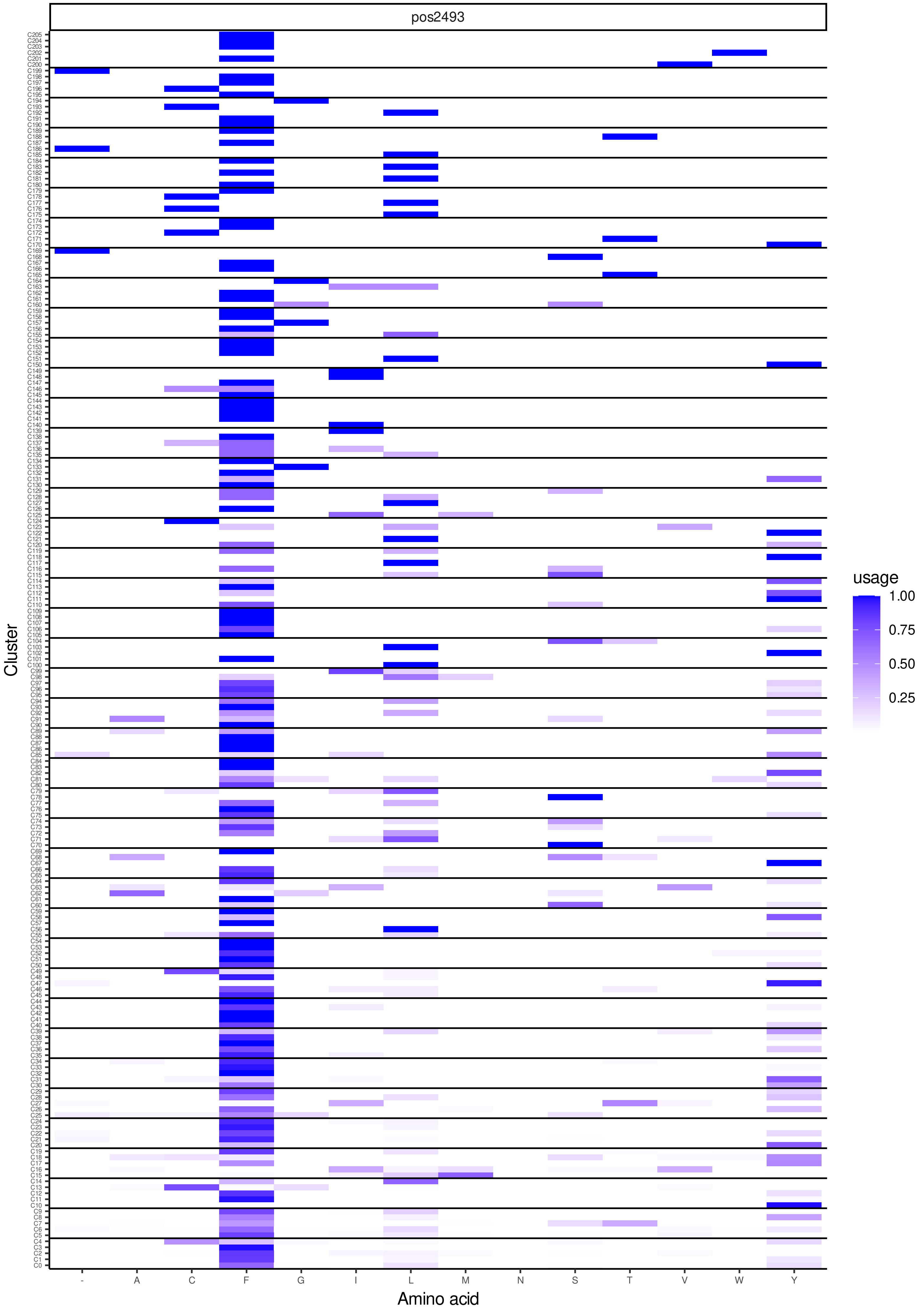
pos2493
Cluster
Amino acid
- A C F G I L M N S T V W Y
usage
1.00
0.75
0.50
0.25

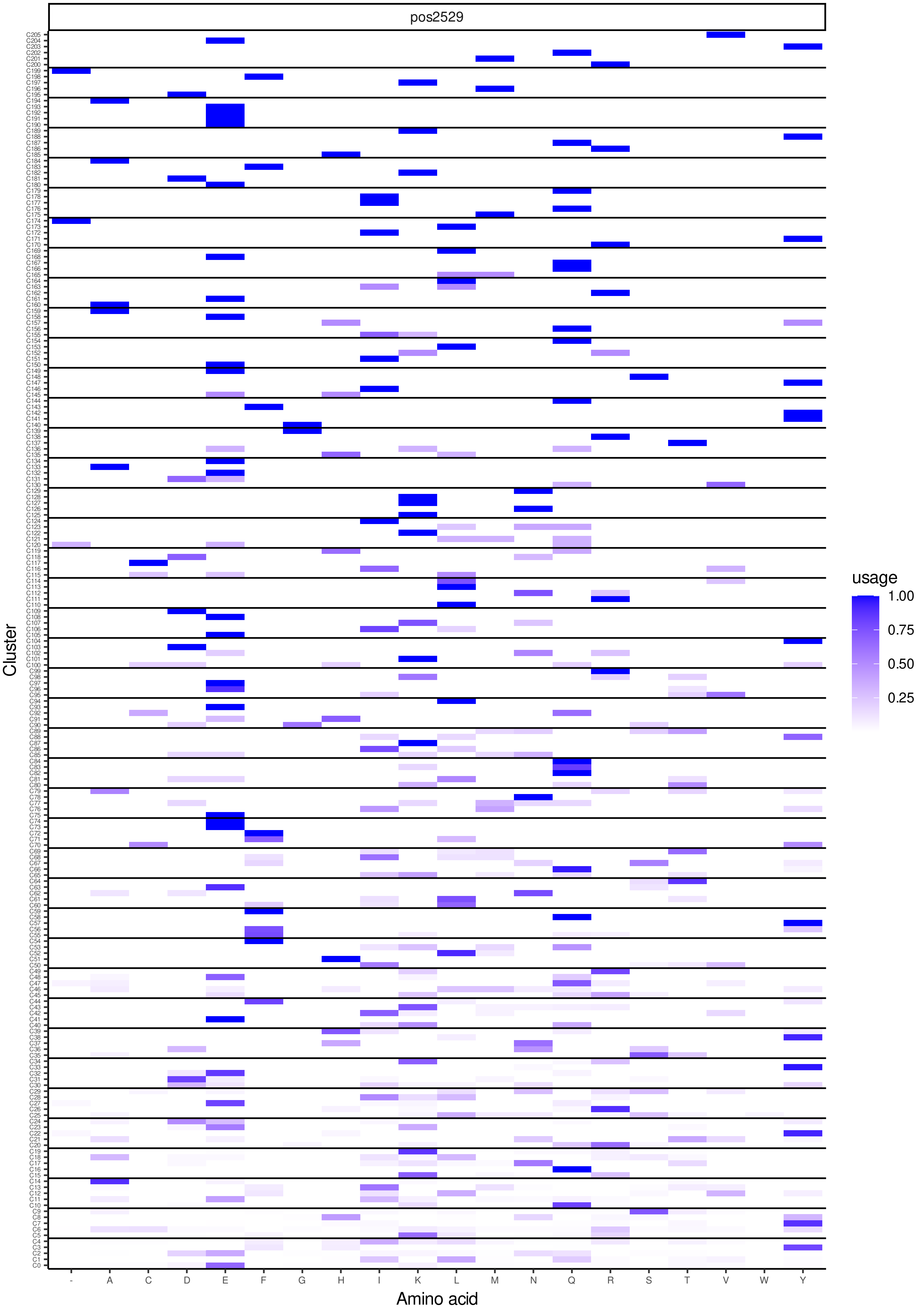

pos2529
Cluster
Amino acid
usage
1.00
0.75
0.50
0.25
- A C D E F G H I K L M N Q R S T V W Y

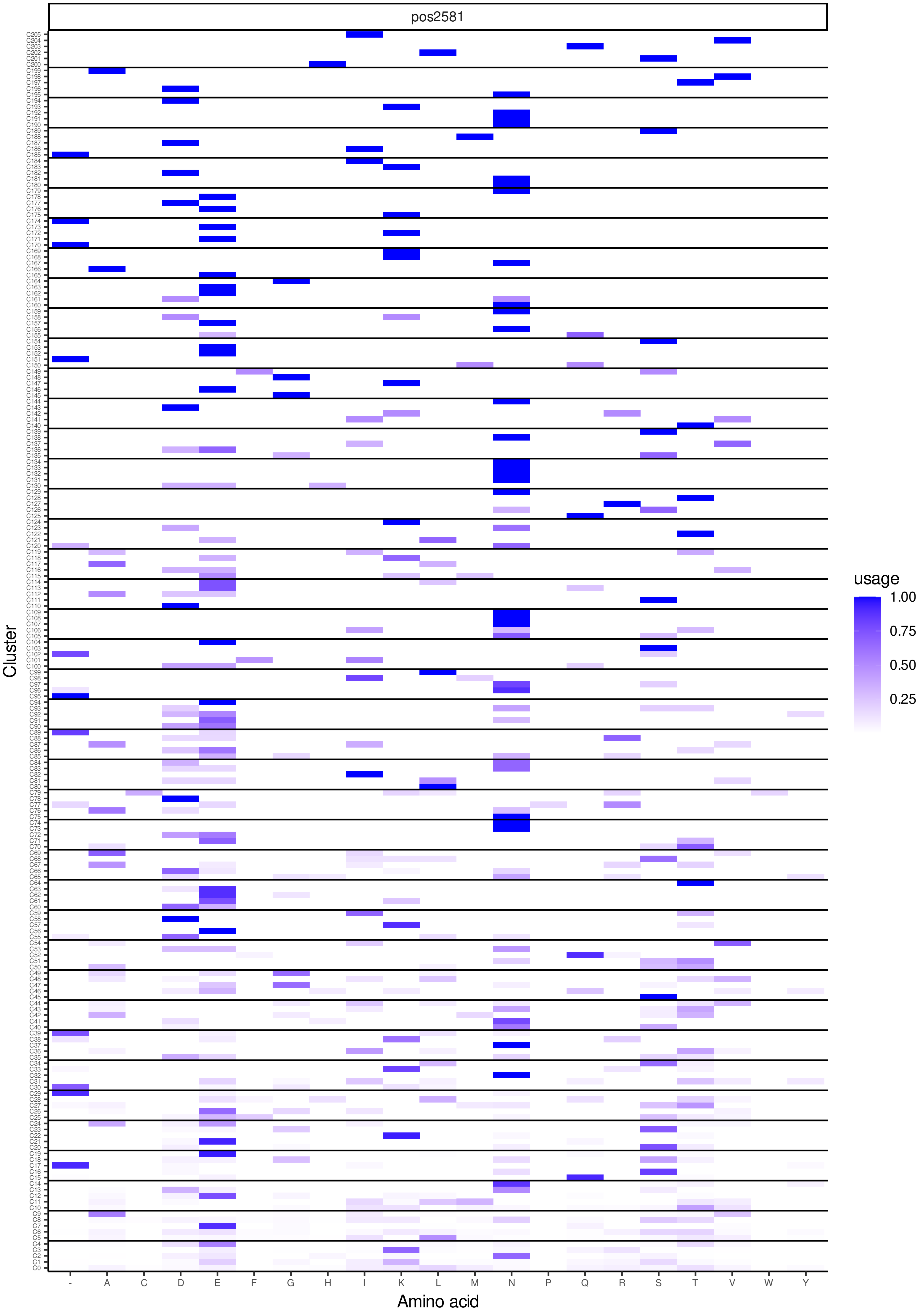
pos2581
Cluster
Amino acid
usage
1.00
0.75
0.50
0.25

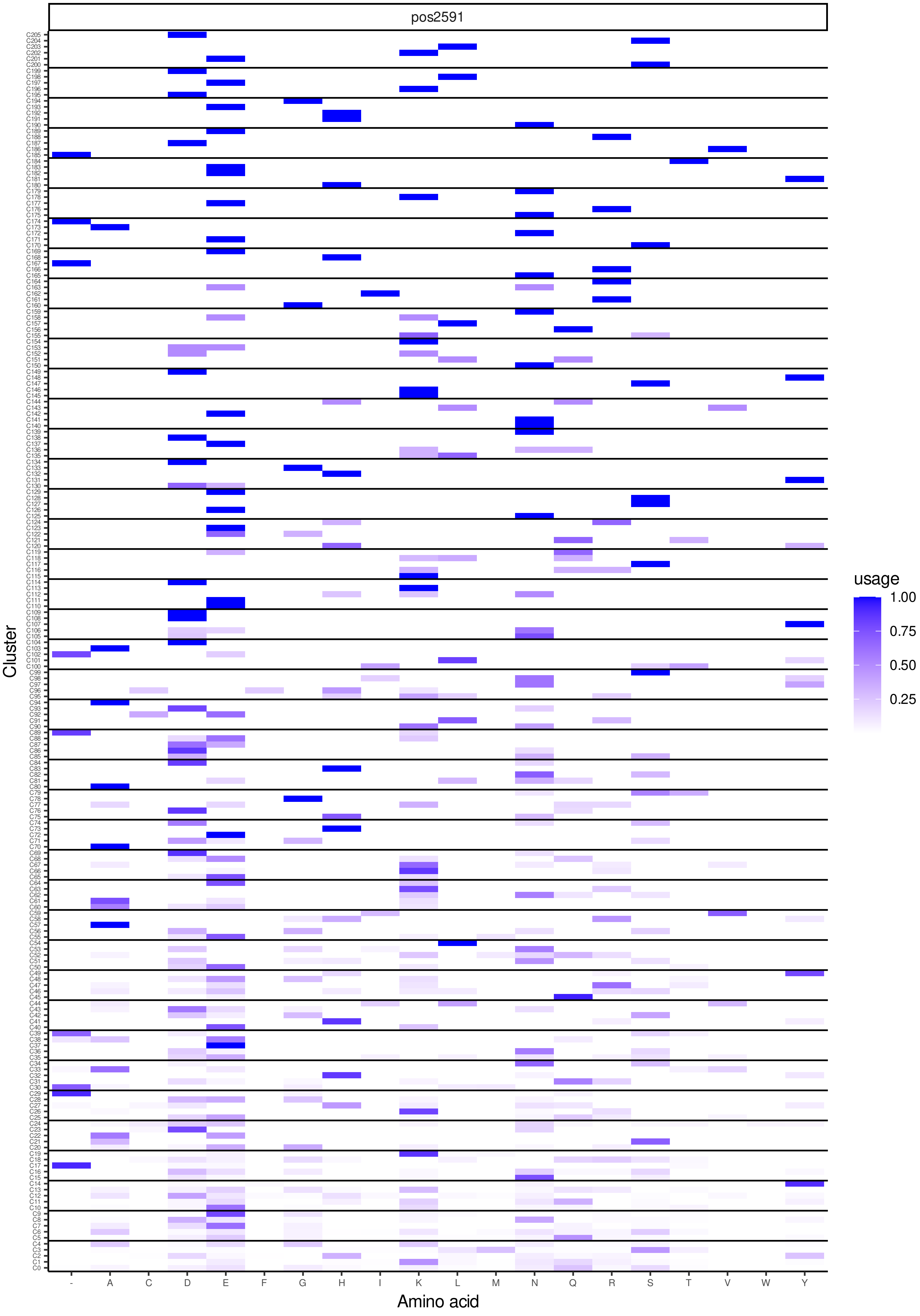
pos2591
Cluster
Amino acid
- A C D E F G H I K L M N Q R S T V W Y
usage
1.00
0.75
0.50
0.25

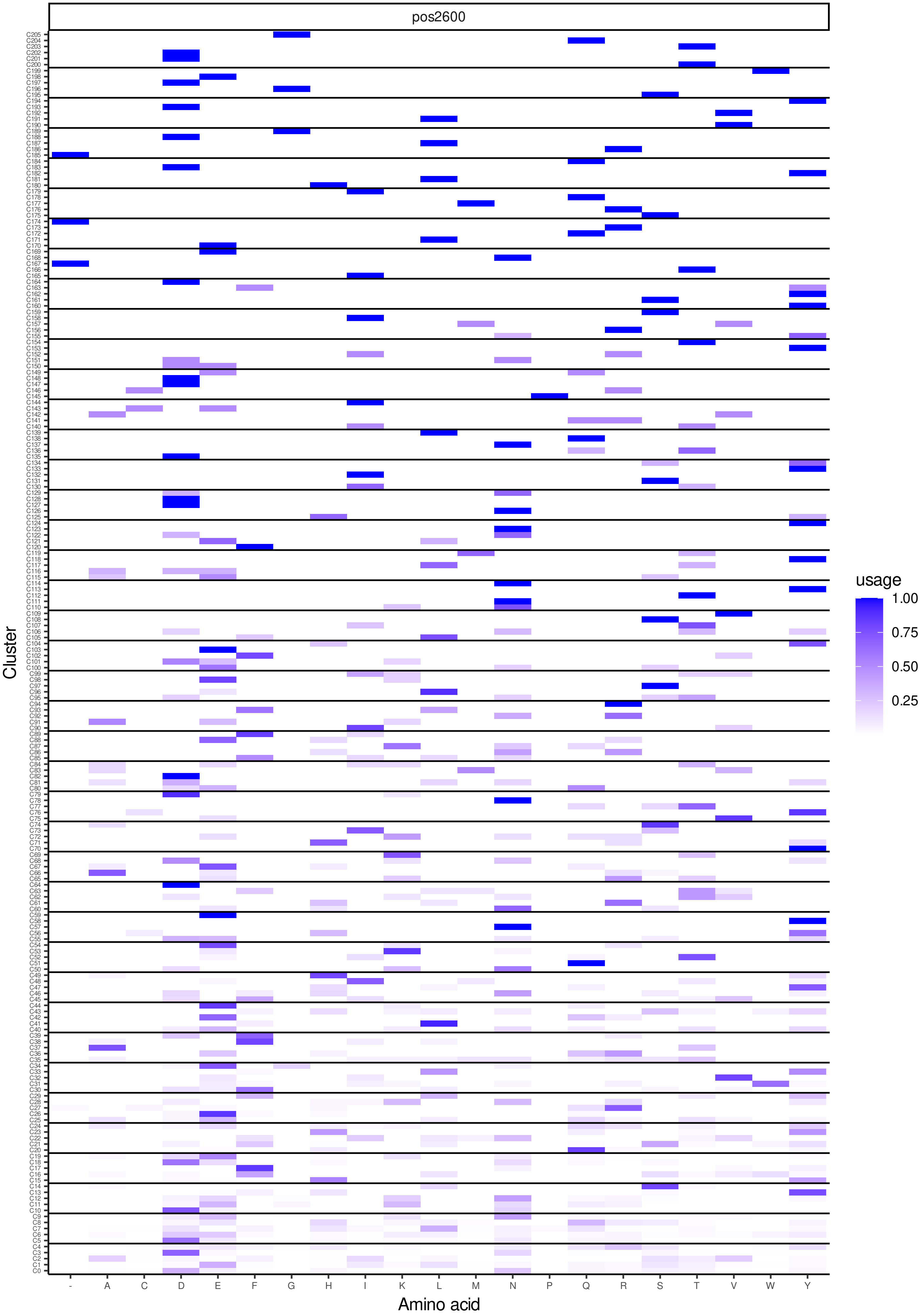
pos2600
Cluster
Amino acid
- A C D E F G H I K L M N P Q R S T V W Y
usage
1.00
0.75
0.50
0.25

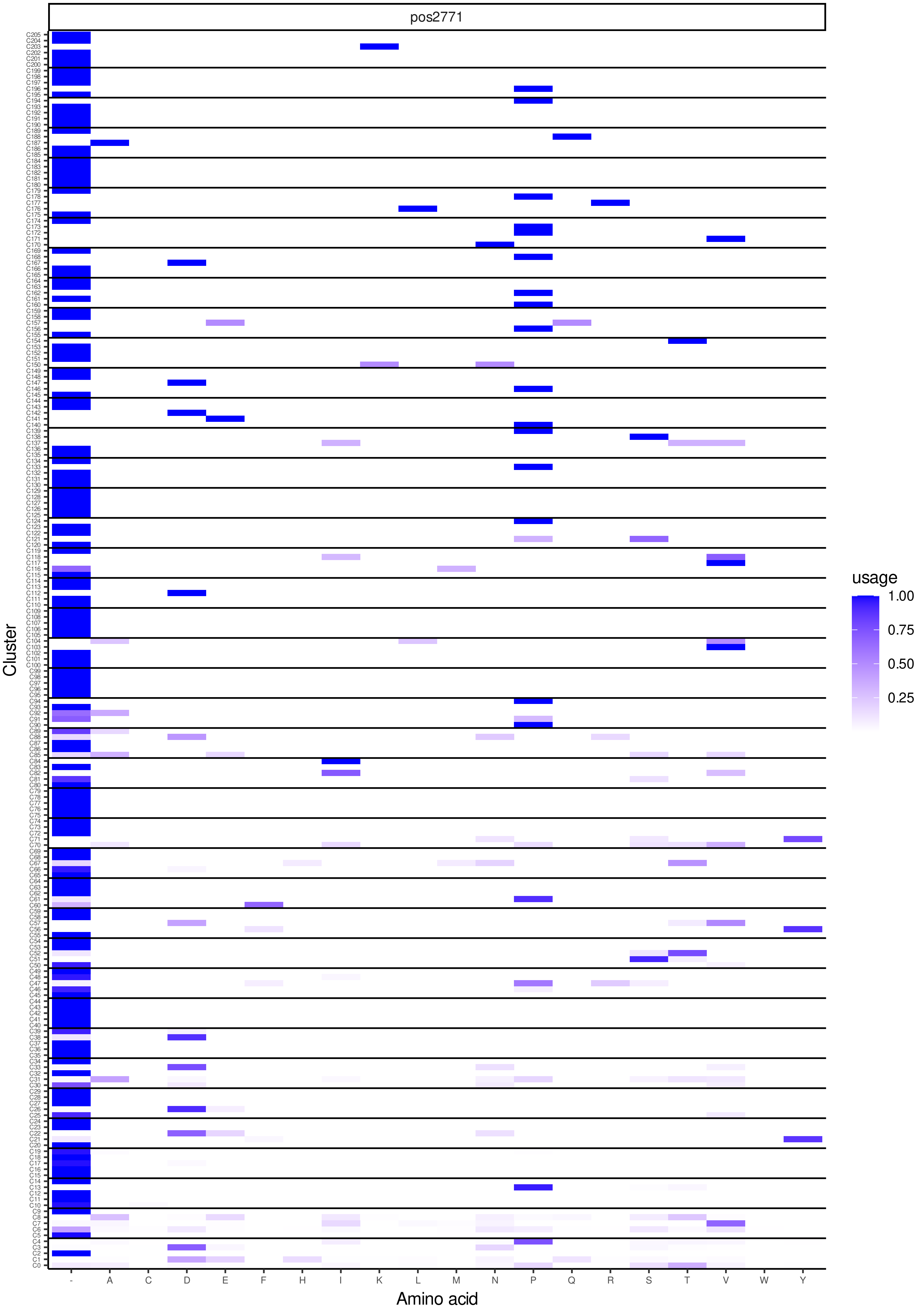
pos2771
Cluster
Amino acid
usage
1.00
0.75
0.50
0.25
- A C D E F H I K L M N P Q R S T V W Y

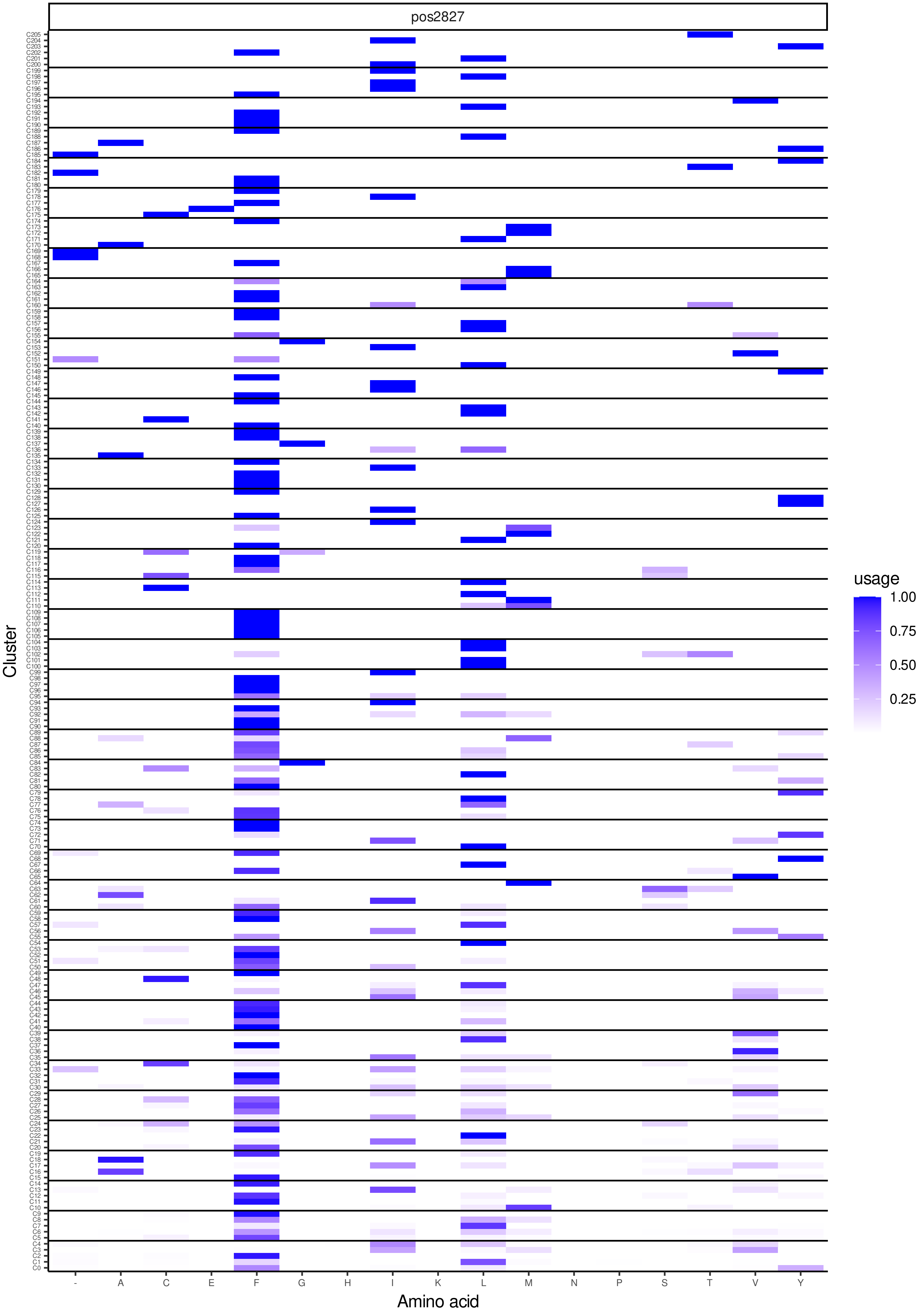
pos2827
Cluster
Amino acid
- A C E F G H I K L M N P S T V Y
usage
1.00
0.75
0.50
0.25
