# Supplementary material for: Annotation and Analysis of 3902 Odorant Receptor Protein Sequences from 21 Insect Species Provide Insights into the Evolution of Odorant Receptor Gene Families in Solitary and Social Insects
Source: Genes (Basel). 2022 May 20;13(5):919. doi: 10.3390/genes13050919 (PMC9141868; doi:10.3390/genes13050919)
Supplement: Supplementary file 1 [file Supplementary Figure S1.pdf › SupplementaryFiles_OR/Supplementary Figure S1.pdf]

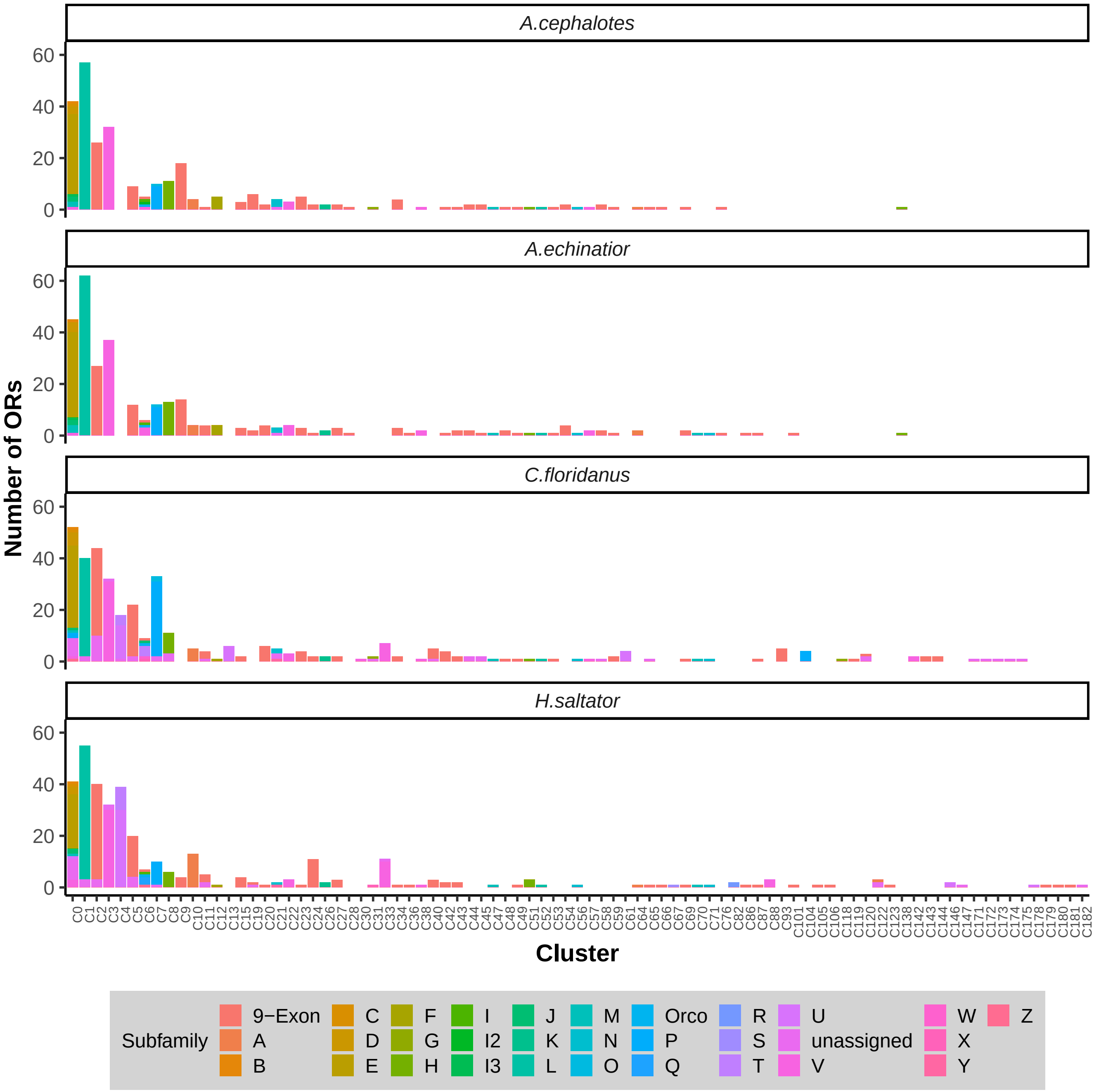
A.cephalotes
A.echinatior
C.floridanus
H.saltator
Number of ORs
0
20
40
60
Cluster
C0 C1 C2 C3 C4 C5 C6 C7 C8 C9 C10 C11 C12 C13 C15 C19 C20 C21 C22 C23 C24 C26 C27 C28 C30 C31 C33 C34 C36 C38 C40 C42 C43 C44 C45 C47 C48 C49 C51 C52 C53 C54 C56 C57 C58 C59 C61 C64 C65 C66 C67 C69 C70 C71 C76 C82 C86 C87 C88 C93 C101 C104 C105 C106 C118 C119 C120 C122 C123 C138 C142 C143 C144 C146 C147 C171 C172 C173 C174 C175 C178 C179 C180 C181 C182
Subfamily
9−Exon
A
B
C
D
E
F
G
H
I
I2
I3
J
K
L
M
N
O
Orco
P
Q
R
S
T
U
unassigned
V
W
X
Y
Z
